# Electro-reduction of carbon dioxide at low over-potential at a metal–organic framework decorated cathode

**DOI:** 10.1038/s41467-020-19236-4

**Published:** 2020-10-29

**Authors:** Xinchen Kang, Lili Li, Alena Sheveleva, Xue Han, Jiangnan Li, Lifei Liu, Floriana Tuna, Eric J. L. McInnes, Buxing Han, Sihai Yang, Martin Schröder

**Affiliations:** 1 Department of Chemistry, University of Manchester, Manchester, M13 9PL UK; 2 Beijing National Laboratory for Molecular Sciences, CAS Key Laboratory of Colloid, Interface and Chemical Thermodynamics, Institute of Chemistry, Chinese Academy of Science, 100190 Beijing, China; 3 Photon Science Institute, University of Manchester, Manchester, M13 9PL UK

**Keywords:** Heterogeneous catalysis, Metal-organic frameworks

## Abstract

Electrochemical reduction of carbon dioxide is a clean and highly attractive strategy for the production of organic products. However, this is hindered severely by the high negative potential required to activate carbon dioxide. Here, we report the preparation of a copper-electrode onto which the porous metal–organic framework [Cu_2_(L)] [H_4_L = 4,4′,4″,4′′′-(1,4-phenylenebis(pyridine-4,2,6-triyl))tetrabenzoic acid] can be deposited by electro-synthesis templated by an ionic liquid. This decorated electrode shows a remarkable onset potential for reduction of carbon dioxide to formic acid at −1.45 V vs. Ag/Ag^+^, representing a low value for electro-reduction of carbon dioxide in an organic electrolyte. A current density of 65.8 mA·cm^−2^ at −1.8 V vs. Ag/Ag^+^ is observed with a Faradaic efficiency to formic acid of 90.5%. Electron paramagnetic resonance spectroscopy confirms that the templated electro-synthesis affords structural defects in the metal–organic framework film comprising uncoupled Cu(II) centres homogenously distributed throughout. These active sites promote catalytic performance as confirmed by computational modelling.

## Abstract (web summary)

Electrochemical reduction of carbon dioxide is a highly attractive strategy for the production of organic products of economic value. Here, the authors report the electrochemical reduction of carbon dioxide to formic acid over a copper-based metal–organic framework decorated electrode at low over-potential.

## Introduction

Increasing CO_2_ levels in the atmosphere present significant environmental impacts^1–3^. Thus, routes to carbon capture and storage, as well as development of CO_2_ reduction technologies to afford chemical feedstocks are being developed^4–6^. Reduction of CO_2_ is economically and environmentally desirable but technically challenging because the high energy of the C=O bond (750 kJ·mol^−1^) makes it an extremely stable molecule that is reluctant to transform^7–9^. One effective method to overcome the high activation barrier is through electrochemical catalytic reduction in which CO_2_ is reduced under mild conditions^10–13^. Copper-based electrodes have been reported as powerful catalysts for the electro-reduction of CO_2_ to give numerous products including CO, formic acid and, recently, C_2_ and other products^14–18^. Formic acid is heavily used in chemical industries for leather, dyeing and textiles, and can be converted directly within fuel cells^13,19^,  and is therefore a valuable C_1_ product. Reduction of CO_2_ at Cu-based electrodes can proceed via O-bound HCOO– or C-bound –COOH intermediates that are formed from electrochemically generated ·CO_2_^−^ radicals. The latter route can diverge to other possible products^20,21^, but the electro-reduction of CO_2_ via an O-bound HCOO– pathway may enhance the Faradaic efficiency for the formation of formic acid. In addition, the formation of formic acid requires a highly negative potential for CO_2_ reduction, and this often leads to decomposition of copper-based catalyst via reduction of metal sites during electrolysis. Although emerging electrocatalysts have been reported for reduction of CO_2_ to formic acid at low over-potentials^17^, more work needs to be undertaken to understand the details of this catalysis^22,23^.

In recent years, there has been much interest in metal–organic framework (MOF) materials as crystalline porous hosts for gas adsorption, separation and catalysis^24–26^. Their high surface area, potentially active metal centres, diverse pore structure and, in exceptional cases, their redox activity, promote their use as electrocatalysts for H_2_ and O_2_ evolution and reduction of O_2_^27–29^. However, highly crystalline MOFs with fully coordinated metal centres exhibit only low charge-transfer ability and formally no active centres for electrocatalysis^12^. Therefore, in order to drive efficient electrocatalysis, MOFs that exhibit high capacity for charge-transfer and incorporate accessible metal sites are highly desirable.

Herein, we report the templated electrochemical growth of the Cu(II) complex [Cu_2_(L)] [H_4_L = 4,4′,4″,4′′′-(1,4-phenylenebis(pyridine-4,2,6-triyl))tetrabenzoic acid] (Supplementary Fig. 1) on a Cu-foam electrode to introduce abundant structural defects comprised of active Cu(II) sites within the deposited film. The resultant electrode, Cu_2_(L)-e/Cu, shows excellent activity for the reduction of CO_2_ to formic acid with an onset potential of −1.45 V vs. Ag/Ag^+^ for production of formic acid, a Faradaic efficiency (FE_HCOOH_) reaching 90.5% at −1.8 V vs. Ag/Ag^+^ and a current density of 65.8 mA·cm^−2^. Side reactions, such as H_2_ evolution and reduction of Cu(II) sites, are effectively hindered at low potentials, and the catalytic mechanism has been studied by electron paramagnetic resonance (EPR) spectroscopy and density functional theory (DFT) calculations.

## Results and discussion

### Electrosynthesis of Cu_2_(L)-e/Cu

**Fig. 1:**
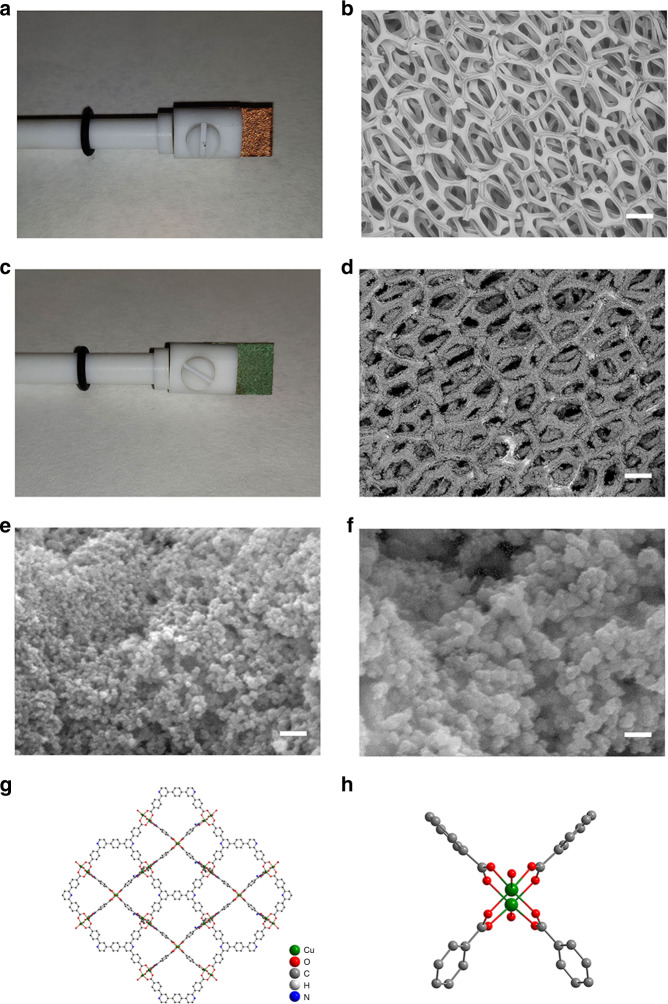
Images and morphology. **a**, **b** Photograph (**a**) and SEM image (**b**) of a fresh Cu-foam electrode (0.5 × 1.0 cm^2^). **c** Photograph of the as-prepared electrode Cu_2_(L)-e/Cu (0.5 × 1.0 cm^2^). **d**–**f** SEM images of the as-prepared electrode Cu_2_(L)-e/Cu. **g**–**h** Views of the crystal structure of [Cu_2_(L)] (CCDC 1531622)^31^. Hydrogen atoms are omitted for clarity. The scale bars of **b**, **d**–**f** are 300 µm, 300 µm, 300 nm and 100 nm, respectively.

The ligand H_4_L was synthesised via a three-step method from our previous report^30^. The electrode, Cu_2_(L)-e/Cu, was prepared by electro-synthesis of the MOF on Cu-foam at a potential of 8.0 V at 60 °C in the presence of the ionic liquid 1-ethyl-3-methylimidazolium acetate (EmimOAc, Supplementary Fig. 2) as supporting electrolyte (Supplementary Fig. 3). Upon completion, the porous Cu-foam was uniformly coated with green crystallites of Cu_2_(L)-e (Fig. 1a–d) thus limiting the further supply of Cu^2+^ ions from the anode for synthesis of more MOF material. SEM images confirm the spherical morphology of the complex film with an average particle size of ~50 nm (Fig. 1e, f). For comparison, the MOF was also synthesised by a conventional solvothermal reaction [denoted as Cu_2_(L)-t]^31^, which crystallised in much larger octahedral-shaped crystals of several microns in size (Supplementary Fig. 4). The structure of Cu_2_(L) comprises of a 3D network built around binuclear [Cu_2_(OOCR)_4_] paddlewheels with four bridging carboxylate ligands (Fig. 1g, h)^32^.

### Structural analysis and characterisation of Cu_2_(L)

**Fig. 2:**
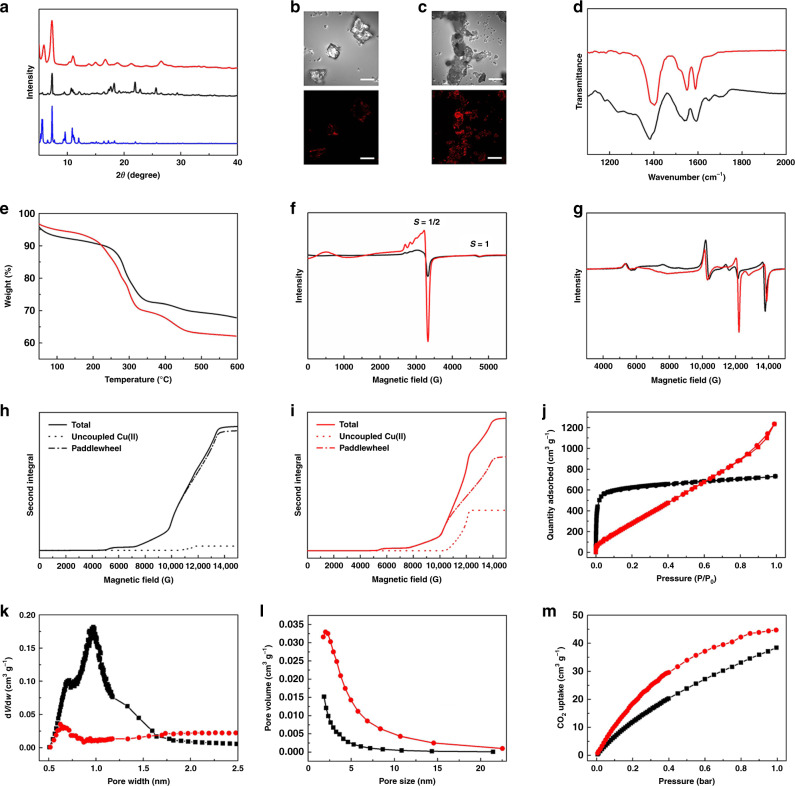
Characterisations of Cu_2_(L)-t (black lines) and Cu_2_(L)-e (red lines). **a** PXRD patterns (blue line refers to simulated PXRD pattern). The defects in Cu_2_(L)-e result in broadening of peaks due to poorer crystallinity of the material. **b** Micrograph (top) and CFM image (bottom) of Cu_2_(L)-t. The fluorescence (shown in red colour) indicates the presence of crystal defects determined by the oligomerisation of furfuryl alcohol. **c** Micrograph (top) and CFM image (bottom) of Cu_2_(L)-e. **d** Selected FTIR spectra (full spectra are shown in Supplementary Information). **e** TGA curves. **f** X-band EPR spectra at room temperature. **g** Q-band EPR spectra at room temperature. **h** Second integrals from simulated Q-band EPR spectra for Cu_2_(L)-t. **i** Second integrals from simulated Q-band EPR spectra for Cu_2_(L)-e. **j** N_2_ adsorption/desorption isotherms at 77 K. **k** Micropore size distribution. **l** Mesopore size distribution. **m** Adsorption isotherms for CO_2_ at 298 K (desorption data are shown in Supplementary Information). Data for HKUST-1-e are shown in Supplementary Information. The scale bars of **b** and **c** are 20 μm.

Power X-ray diffraction (PXRD) confirmed that Cu_2_(L)-t and Cu_2_(L)-e (removed from the electrode) have the same structure consistent with the simulated pattern (Fig. 2a)^31^. We sought to characterise the nature of the structural defects in Cu_2_(L)-e, which can be captured vividly by confocal fluorescence microscopy (CFM) using the oligomerisation of furfuryl alcohol as a probe^33^. In this case, open Cu(II)-site based defects act as Lewis acid centres that catalyse the formation of oligomers of furfuryl alcohol to generate strong fluorescence. The microphotographs and CFM images show (Fig. 2b, c) fluorescence evenly across particles of Cu_2_(L)-e, whereas only the surfaces of Cu_2_(L)-t exhibit fluorescence, confirming the wide distribution of open Cu(II) defect sites throughout Cu_2_(L)-e. Fourier transform infrared (FTIR) spectroscopy shows that the υ(C=O) stretching vibration of the carboxylate group in Cu_2_(L)-e is blue shifted by 17 cm^−1^, from 1385 cm^−1^ to 1402 cm^−1^, compared to Cu_2_(L)-t, consistent with increased amounts of unbound carboxylate groups in the former (Fig. 2d). A slight decrease in decomposition temperature for Cu_2_(L)-e compared to Cu_2_(L)-t by TGA analysis suggests that the defect structure of Cu_2_(L)-e has slightly reduced thermal stability (Fig. 2e). Full elemental analysis of these materials reveals a Cu:L ratio of 2.4 in Cu_2_(L)-e (Supplementary Table 1), higher than the 2.0 ratio obtained for Cu_2_(L)-t. This reflects the direct growth of Cu_2_(L)-e onto the Cu-foam surface to give a more Cu-rich environment. X-band and Q-band EPR spectra at room temperature reveal a significantly greater concentration of uncoupled Cu(II) defect sites in Cu_2_(L)-e than in Cu_2_(L)-t (Fig. 2f, g, Supplementary Figs. 5–8). The spectra are dominated by the characteristic spin triplet spectrum of the [Cu_2_(OOCR)_4_] paddlewheel structure, which arise from strong antiferromagnetic exchange (singlet-triplet gap *ca*. 300 cm^−1^) within the binuclear moiety and the thermal population of the excited *S* = 1 state, which has a zero-field splitting of *ca*. 0.3 cm^−1^. The characteristic forbidden transition (*m*_S_ = ±2) associated with the triplet spin state of the Cu_2_ entity is clearly observed (*ca*. 5300 G) in all Q-band spectra of Cu_2_(L)-e and Cu_2_(L)-t (Fig. 2g and Supplementary Fig. 8). In addition, features due to uncoupled Cu(II) (*S* = ½, *g*_xx,yy_ = 2.07, *g*_zz_ = 2.32, *A*_xx,yy_^Cu^ = 33.6 MHz, and *A*_zz_^Cu^ = 450 MHz) ions are observed (*ca*. 3250G at X-band; 12000G at Q-band), and these signals are more pronounced in Cu_2_(L)-e than in Cu_2_(L)-t (Fig. 2f, g). The relative concentration of [Cu_2_(OOCR)_4_] paddlewheel units and uncoupled free Cu(II) is given by the relative intensity of the second integral of simulations of the Q-band EPR spectra, weighted by the room temperature Boltzmann population of the spin triplet state of the [Cu_2_(OOCR)_4_] paddlewheel (Fig. 2h, i and Supplementary Table 2)^34–36^. Using this approach it was determined that Cu_2_(L)-t and Cu_2_(L)-e contain 1.5% and 15.3% of uncoupled Cu(II) sites, respectively, confirming that electro-synthesis generates an order of magnitude greater number of defect sites.

### Analysis of porosity in Cu_2_(L)

The porosity of these MOFs was studied by N_2_ isotherms at 77 K (Fig. 2j and Supplementary Table 1). Desolvated Cu_2_(L)-t shows a Type-I sorption profile consistent with the expected uniform microporosity. In contrast, desolvated Cu_2_(L)-e exhibits a profile between Type-I and Type-IV, suggesting the presence of both mesopores and micropores. The distribution of micro- and mesopores within both materials has been analysed by Horvath-Kawazoe and Barrett–Joyner–Halenda (BJH) methods, respectively (Fig. 2k, l). The reduction in micropores in Cu_2_(L)-e results in a low N_2_ uptake at low pressure, while Cu_2_(L)-e, generated by the template-effect of the ionic liquid during electro-synthesis^33^, shows a total pore volume of 1.89 cm^3^·g^−1^, significantly larger than that of Cu_2_(L)-t (0.32 cm^3^·g^−1^), reflecting the presence of mesopores in Cu_2_(L)-e. CO_2_ adsorption in desolvated Cu_2_(L)-t and Cu_2_(L)-e at 1.0 bar and 298 K are 38.5 and 44.7 cm^3^ g^−1^, respectively (Fig. 2m). Higher isosteric heats of adsorption (*Q*_*st*_) were obtained for Cu_2_(L)-e (Supplementary Figs. 9, 10), suggesting stronger interactions with CO_2_ than in Cu_2_(L)-t, consistent with the presence of additional active sites in the former. In summary, electro-synthesised Cu_2_(L)-e can be grown directly onto Cu-foam to afford a decorated electrode incorporating a defect structure with active Cu(II) sites that show strong interaction with CO_2_, a key feature for an optimal catalyst for CO_2_ reduction^37^. For comparison, the benchmark system HKUST-1 has also been electro-synthesised onto Cu-foam (denoted as HKUST-1-e/Cu) using the same method, and fully characterised by SEM, PXRD, TGA and BET (Supplementary Figs. 11–14 and Supplementary Table 1). High crystallinity and large particle sizes (~1 μm) were obtained for HKUST-1-e, consistent with its facile crystal growth.

### Electrochemical reduction of CO_2_

**Fig. 3:**
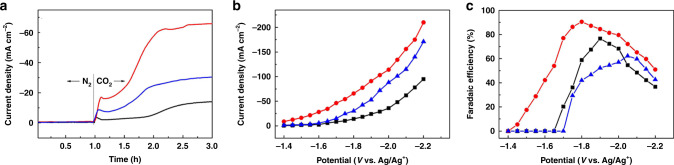
Electrochemical reduction of CO_2_ over Cu_2_(L)-t/CP (black lines), Cu_2_(L)-e/Cu (red lines), and HKUST-1-e/Cu (blue lines). **a** Plot of current density vs. time. **b** Plot of current density *vs*. potential. **c** Plot of FE_HCOOH_ vs. potential.

To test performance for electrochemical reduction of CO_2_, Cu_2_(L)-e/Cu and HKUST-1-e/Cu were used directly as electrodes, while a powder sample of Cu_2_(L)-t was loaded onto carbon paper (CP) with Nafion-D521 as adhesive to fabricate a Cu_2_(L)-t/CP electrode^12^. All experiments were performed in an H-type cell with 0.5 M 1-ethyl-3-methylimidazolium tetrafluoroborate (EmimBF_4_) in acetonitrile (0.5 M EmimBF_4_/MeCN) as catholyte. As shown from the linear sweep voltammetry at the Cu_2_(L)-e/Cu electrode, negligible current was generated in N_2_-saturated electrolyte, while the current density increased dramatically in CO_2_-saturated electrolyte, confirming a strong response to CO_2_ (Supplementary Fig. 15). The dependence of current density on time (*i*-*t* curves) for all three electrodes is shown in Fig. 3a. Minimal current density was observed with N_2_, whereas a rise in current density is observed on introduction of CO_2_. The current density continuously increases in the presence of CO_2_ and stabilises after 1 h for the three electrodes. The Cu_2_(L)-e/Cu electrode shows the highest current density, and for all systems, formic acid was found to be the only carbon-containing product based on GC and ^1^H NMR spectroscopic analysis of gas and liquid phases, respectively, after electrolysis. No carbon-containing product was detected in the absence of CO_2_ from the catholyte, confirming that the generated formic acid is derived solely from introduced CO_2_ rather than decomposition of the catalyst or electrolyte. The current density and Faradaic efficiency for formation of formic acid (FE_HCOOH_) were recorded after 2 h of electrolysis (Fig. 3b, c) from −1.4 V to −2.2 V vs. Ag/Ag^+^. H_2_ is the only bi-product within this potential range. Significantly, the Cu_2_(L)-e/Cu electrode shows an onset potential of −1.45 V vs. Ag/Ag^+^ for the generation of formic acid, representing one of the lowest potential observed in organic electrolytes for systems reported to date (Supplementary Table 3)^6,38–40^, although a recent Cu-porphyrin system shows an onset voltage as low as −1.4 V vs. Ag/Ag^+^.^17^ The current density increases with increasing negative potential, but FE_HCOOH_ increases and then decreases, reaching a maximum of 90.5% at −1.8 V vs*.* Ag/Ag^+^ with a current density of 65.8 mA·cm^−2^. The value of FE_HCOOH_ is maintained at >80% between −1.75 V to −1.95 V vs. Ag/Ag^+^. In comparison, the Cu_2_(L)-t/CP electrode exhibits a higher onset potential of −1.7 V vs. Ag/Ag^+^ for formic acid production and a lower current density across the potential range, with a maximum FE_HCOOH_ of 77% observed at −1.9 V vs. Ag/Ag^+^. For the HKUST-1-e/Cu electrode, an even higher onset potential of −1.75 V vs. Ag/Ag^+^ was observed for production of formic acid, and FE_HCOOH_ reaches a maximum of 62% at −2.05 vs. Ag/Ag^+^. Thus, Cu_2_(L)-e/Cu demonstrates an excellent performance for electro-reduction of CO_2_ in terms of the low onset potential and high FE_HCOOH_ with relatively low over-potentials. The current density for formic acid production increases rapidly from the onset potential to −2.0 V vs. Ag/Ag^+^, and the production of formic acid progresses slowly thereafter (Supplementary Fig. 16), reflecting the enhanced production of H_2_ at potentials more negative than −2.0 V vs. Ag/Ag^+^. At potentials more negative than −2.0 V vs. Ag/Ag^+^, the HKUST-1-e/Cu electrode shows a rise in current density (Fig. 3b) with the current density of formic acid reaching a plateau (Supplementary Fig. 16). An undecorated Cu-foam electrode (Fig. 1a, b) was also tested, and a current density of 4.2 mA·cm^−2^ with a FE_HCOOH_ of 20.5% after 2 h electrolysis at −1.8 V vs. Ag/Ag^+^ were observed (Supplementary Fig. 17), demonstrating the critical role of defect Cu_2_(L) on the performance of the electrode. It should be noted that a nanostructured Cu-foam has been explored previously as an electrocatalyst^16^; the Cu-foam in the current study is of a different type and is much smoother.

Upon completion of reaction, HKUST-1-e turned amorphous and partially peeled off the Cu-foam as confirmed by SEM images. In contrast, Cu_2_(L)-e/Cu generally maintained its morphology with a slight aggregation of particles (Supplementary Fig. 18). Thus, the thin coating with small crystallites in the Cu_2_(L)-e/Cu electrode affords chemical and mechanical stability for electrolysis, although like many Cu(II) MOFs^15^, the Cu_2_(L)-e/Cu electrode is unstable in water.

### Variations of EPR spectra of electrodes over time

**Fig. 4:**
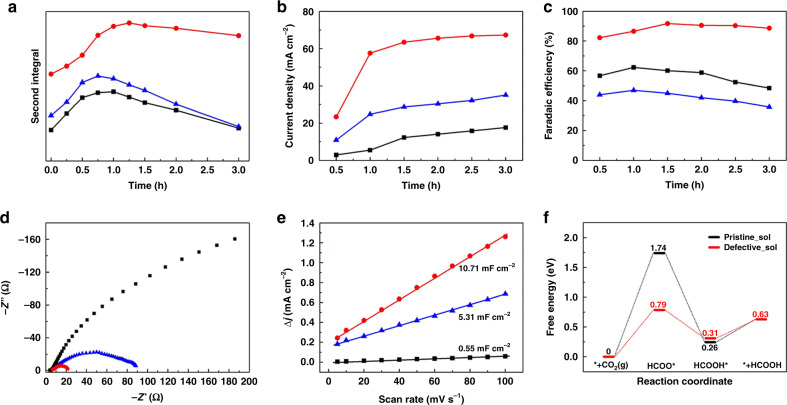
Comparisons of Cu_2_(L)-t/CP (black lines), Cu_2_(L)-e/Cu (red lines) and HKUST-1-e/Cu (blue lines). **a**, Plot of second integral of the X-band EPR signals for uncoupled Cu(II) centres at room temperature vs. time of CO_2_ electrolysis. **b** Plot of current density vs. time of CO_2_ electrolysis at −1.8 V vs. Ag/Ag^+^. **c** Plot of FE_HCOOH_ vs. time of CO_2_ electrolysis at −1.8 V vs. Ag/Ag^+^. **d** Nyquist plots for reduction of CO_2_. **e** Plot of difference in charging current density *vs*. scan rates. **f** DFT calculation of *Gibbs* free energy of electro-reduction of CO_2_ to formic acid over pristine (black lines) and defect Cu_2_(L)-t (red lines) after consideration of solvation effects.

To understand further the mechanism of catalysis of these electrodes, EPR spectra were measured over time intervals of 15 mins during electrolysis of CO_2_ at −1.8 V vs. Ag/Ag^+^. We sought to use EPR spectroscopy to probe changes in concentrations of free, uncoupled Cu(II) centres as the reaction proceeds^41^, and this can be monitored by the relative intensity of the second integral of the EPR spectrum (Fig. 4a). As confirmed above, as-prepared Cu_2_(L)-e/Cu has a high concentration of uncoupled Cu(II) centres due to its defect structure. All three electrodes show an increase in uncoupled Cu(II) content during electrolysis: this is presumably caused by reduction of the [Cu_2_(OOCR)_4_] paddlewheel units under the electrolytic conditions, with subsequent structural disruption. The amount of free Cu(II) sites was maximised after electrolysis for 75, 60 and 45 mins for Cu_2_(L)-e/Cu, Cu_2_(L)-t/CP and HKUST-1-e/Cu, respectively, followed by a slight decrease for Cu_2_(L)-e/Cu and a greater decrease in Cu_2_(L)-t/CP and HKUST-1-e/Cu. The quantities of uncoupled Cu(II) sites decrease after a maximum and are likely due to reduction to diamagnetic (3d^10^) Cu(I) over time (Supplementary Fig. 19), as confirmed by XPS and Auger spectra (Supplementary Fig. 20). These results also support the thesis that free Cu(II) defect sites are responsible for the high current density and FE_HCOOH_ observed for Cu_2_(L)-e/Cu throughout the electrolysis of CO_2_ (Fig. 4b, c). H_2_ evolution becomes more evident over time using Cu_2_(L)-t/CP and HKUST-1-e/Cu electrodes also likely due to generation of Cu(I) sites over time (Supplementary Fig. 21). The reduction of [Cu_2_(OOCR)_4_] paddlewheel MOFs to form Cu(II).Cu(I) species has been reported previously^42^, and is associated with tetrahedral distortion of the reduced metal centre to give an uncoupled Cu(II) centre. The current density for formation of formic acid remains constant after the uncoupled Cu(II) content reaches its maximum value, and so catalysis remains stable up to 5 h (Supplementary Figs. 22, 23). Thus, Cu_2_(L)-e/Cu exhibits the best performance for long-term electrochemical stability for formic acid production. It is notable that the total Faradaic efficiency for formation of formic acid and H_2_ (FE_HCOOH+H2_) is lower than 100% during the first hour of electrolysis (Supplementary Fig. 24), indicating that the electrode evolves during this early period of electrolysis, consistent with the morphological changes observed at the electrode surface (Supplementary Fig. 18).

### Electrochemical characteristics of electrodes

The charge-transfer ability of the electrode also plays an important role in its electrocatalytic performance. As revealed by Nyquist plots at an open circuit potential (Fig. 4d), the Cu_2_(L)-e/Cu, Cu_2_(L)-t/CP and HKUST-1-e/Cu electrodes give charge-transfer resistances (R_ct_) of approximately 17, 114 and 408 Ω, respectively^12,38^. The significantly lower R_ct_ for Cu_2_(L)-e/Cu is most likely due to its microscopic morphology as revealed from SEM images. Thus, nanoparticles of Cu_2_(L) form a compact thin coating on the Cu-foam surface with the overall porous network preserved, whereas rapid crystallisation of HKUST-1 results in bigger crystals and a thick coating of HKUST-1-e/Cu, leading to a higher overall resistance. The poorly-conductive MOF islands on the HKUST-1-e/Cu electrodes thus cause barriers for charge-transfer, in sharp contrast to Cu_2_(L)-e/Cu, where all MOF particles are in close contact with the highly conductive Cu-foam. The interface between Cu_2_(L)-t and CP results in the poor charge-transfer as observed in many other MOF-based electrodes^12^. The double-layer capacitance (*C*_dl_) of the three electrodes was analysed by measuring the capacitive current associated with double-layer charging using the scan-rate dependence of cyclic voltammetric stripping to evaluate the electrochemical active surface area (Fig. 4e)^43^. Cu_2_(L)-e/Cu has the highest value for *C*_dl_ of 10.71 mF·cm^−2^ with values for Cu_2_(L)-t/CP and HKUST-1-e/Cu of 5.31 mF·cm^−2^ and 0.55 mF·cm^−2^, respectively, again evidencing the high active surface area of Cu_2_(L)-e/Cu^44,45^.

### DFT calculations

DFT calculations have been applied widely to uncover the mechanism of electro-reduction of CO_2_^46–48^. The *Gibbs* free energy with respect to potential reaction steps was modelled based upon a [Cu_2_(OOCR)_4_] paddlewheel bound to four 4-(pyridin-2-yl)benzoate groups. Pristine and defect structures representing Cu_2_(L)-t and Cu_2_(L)-e, respectively, were then analysed by DFT calculations with defect Cu_2_(L) modelled with one of the O-donors of one 4-(pyridin-2-yl)benzoate not bound to Cu(II) to afford a vacant site at Cu(II). The corrections for zero point energy and entropy, and the structural model and atomic coordinates of all intermediates are given in Supplementary Tables 4–12. The plausible reaction pathways via C-bound COOH and O-bound HCOO intermediates were analysed (Supplementary Figs. 25, 26) and the effects of solvation were also taken into account (Supplementary Fig. 27). In general, the formation of O-bound HCOO involved a lower *Gibbs* free energy than C-bound COOH or CO over both pristine and defect Cu_2_(L), with formation of O-bound HCOO on defect Cu_2_(L) the most facile pathway leading to selective production of formic acid. In the DFT calculations the free Cu(II) centres in defect Cu_2_(L) are generated by the rupture of one Cu–O(carboxylate) bond, which enables binding of CO_2_ via an O-bound HCOO intermediates. In addition, the defect structure leads to weaker hydrogen bonding between the MOF and product, facilitating the release of HCOOH from the surface of the Cu_2_(L)-e/Cu electrode. The same pattern of *Gibbs* free energy is also observed on inclusion of solvation effects (Supplementary Fig. 27). Figure 4f shows the DFT calculated diagram of *Gibbs* free energy of electro-reduction of CO_2_ to formic acid over pristine and defect Cu_2_(L) with solvation effects considered. Furthermore, both Cu_2_(L)-t and Cu_2_(L)-e electrodes show an increase in uncoupled Cu(II) content during electrolysis (Fig. 4a), consistent with the increasing current density for CO_2_ reduction observed over the first hour of electrolysis. Hydrogen evolution was also interrogated by DFT calculations over pristine and defect Cu_2_(L) (Supplementary Fig. 28) confirming that the defect material will protonate more readily.

### Conclusion

In summary, we have electrochemically prepared the Cu_2_(L)-e/Cu electrode through controllable growth of MOF nanoparticles onto a Cu-foam support. This integrated electrode composed of a thin compact coating of Cu_2_(L) on Cu-foam incorporates uncoupled Cu(II) active sites and shows high conductivity and stability. This electrode shows excellent activity for the electro-reduction of CO_2_ to formic acid, with a low onset potential of −1.45 V vs. Ag/Ag^+^, and the FE_HCOOH_ reaches 90.5% at −1.80 V vs. Ag/Ag^+^ with a current density of 65.8 mA·cm^−2^. Experimental (EPR spectroscopy) and theoretical (DFT) methods confirm that the reaction is driven by defects within the structure of the decorated electrode. In Cu_2_(L)-e/Cu these defects favour reduction of CO_2_ to formic acid versus H_2_ evolution. The facile preparation of Cu-MOF-e/Cu electrodes offers an instructive pathway for the development of other efficient materials that catalyse CO_2_ reduction at less energetic potentials.

## Methods

### Materials

Ionic liquids (ILs), EmimOAc (>99%) and EmimBF_4_ (>99%) were purchased from Lanzhou Yulu Fine Chemical Co., Ltd. Other chemicals were obtained from Sigma-Aldrich Co., UK.

### Ligand synthesis

The ligand H_4_L was prepared by the published procedure^30^. 4-Methylacetophenone (13.4 g, 0.1 mol), terephthalaldehyde (2.70 g, 0.02 mol) and NaOH powder (5.10 g, 0.128 mol) were combined and ground in a ball mill for 90 mins. The resultant solid was dissolved in EtOH (600 mL) with NH_4_OAc (30 g, 0.39 mol), and the solution heated under reflux for 24 h. On cooling the white solid was collected by filtration and recrystallised from toluene to yield white crystals. This product (3.0 g) was combined with an aqueous solution of HNO_3_ (2 M, 36 mL) and heated in an autoclave to 180 °C for 24 h. The reaction mixture was cooled to room temperature and the solid H_4_L was collected and washed with distilled water until the filtrate was pH neutral. The solid was washed further with acetone and dried *in vacuo*.

### Characterisations

The morphology of the materials was characterised on a SEM Quanta 650. PXRD analysis was performed on Rigaku Model D/MAX2500 diffractometer using Cu-Kα radiation at a scan speed of 2^o^/min. Infrared spectra were collected on an iD5 ATR (Attenuated Total Reflection) instrument, and TGA was measured under N_2_ at a flow rate of 10 mL·min^−1^. EPR spectra at X-band and Q-band were recorded using Bruker Micro spectrometers, and the intensity of the EPR signal of different samples was normalised to the sample quantity. The BET surface areas were obtained from N_2_ adsorption/desorption isotherms recorded on a Micromeritics 3-Flex instrument at 77 K. CO_2_ adsorption isotherms were obtained using Micromeritics 3-Flex at 273 K, 283 K and 298 K, and the value for *Q*_*st*_ for CO_2_ adsorption was estimated by fitting these isotherms to the Van t’ Hoff equation. X-ray photoelectron spectroscopy (XPS) was performed on a Kratos Axis Ultra DLD spectrometer with a monochromated Al-Kα X-ray source (*E* = 1486.6 eV, 10 mA emission). Fluorescence micrographs were recorded on an Olympus Fluoview FV-1000 instrument to measure the fluorescence generated by the oligomerisation of 1 mL furfuryl alcohol catalysed by 10 mg of MOF at 60 °C over 2 h.

### Solvothermal synthesis of Cu_2_(L)-t

The solvothermal synthesis of Cu_2_(L)-t was conducted by following the literature method^31^. Cu(NO_3_)_2_·3H_2_O (24.2 mg, 0.10 mmol) and H_4_L (35.6 mg, 0.05 mmol) were dissolved in DMF (3 mL) with 8 M HNO_3_ (0.3 mL). The solution was heated at 80 °C for 48 h in a pressure tube. On cooling to room temperature, the precipitate was collected by centrifugation, washed five times with acetone (10 mL) and dried in vacuo.

### Electro-preparation of Cu_2_(L)-e/Cu

This was performed in a 50 mL single cell using a two-electrode system comprising Cu foam sheets (0.5 cm × 1.0 cm^2^) as both cathode and anode with a solution of DMF/dioxane/H_2_O (2:1:1 v/v/v; 50 mL with 5 drops of aqueous HCl (37%)containing EmimOAc (1.0 mL) as supporting electrolyte. H_4_L (33 mg, 0.05 mmol) was dissolved in the electrolyte solution under stirring to form a homogeneous solution. The electrolysis was undertaken for 10 min at 8.0 V and 60 °C. The as-synthesised MOF-decorated electrode was immersed in a mixture of acetone/acetonitrile (1:1 v/v) and the solvent exchanged five times to remove any residual electrolyte and unreacted materials. The preparation of HKUST-1-e/Cu electrode was undertaken using the same method as used for Cu_2_(L)-e/Cu but using a different ligand (trimesic acid).

### Preparation of electrodes

The Cu_2_(L)-e/Cu and HKUST-1-e/Cu were used directly as electrodes. For the Cu_2_(L)-t/CP electrode, 5.0 mg of Cu_2_(L)-t were suspended in 0.5 mL of isopropanol containing 25 μL of Nafion D-521 dispersion (5 wt%) and treated with ultrasound to form a homogeneous ink. 150 μL of the ink was spread onto the hydrophobic CP (0.5 × 0.5 cm^2^) surface and dried at 60 °C.

### Electrochemical reduction of CO_2_

The electro-reduction of CO_2_ was carried out on an electrochemical workstation (CHI 660E, USA). LSV and electrolysis were conducted in an H-type cell with a three-electrodes configuration consisted of working electrode, CP (0.5 × 0.5 cm^2^) as counter electrode, and Ag/Ag^+^ (0.01 M AgNO_3_ in 0.1 M TBAP-MeCN) as reference electrode^12^. The catholyte and anolyte were 0.5 M EmimBF_4_/MeCN and 0.1 M H_2_SO_4_ aqueous solution, respectively, separated by a Nafion-115 membrane. N_2_ and CO_2_ were bubbled into the catholyte prior to the experiments. For cyclic voltammetric measurements, a scan speed of 20 mV·s^−1^ was used over a potential range from −1.2 V to −2.4 V vs. Ag/Ag^+^.

Electrolysis experiments were performed with an initial CO_2_ flow rate of 20 mL min^−1^ prior to the experiment, which was decreased to 10 mL min^−1^ after the electrolyte was saturated with CO_2_. After electrolysis, the gaseous products were collected using a gas bag and analysed by GC and liquid products measured by ^1^H-NMR spectroscopy. The Faradaic efficiencies (FE) were calculated using the Eq. (1):

1 $${\mathrm{FE}}({\mathrm{\% }}) = \frac{{n_{{\mathrm{product}}} \times n_{{\mathrm{electrons}}} \times F}}{Q} \times 100{\mathrm{\% }}$$

where *n*_product_ is the amount of product (mol) from GC (H_2_) or ^1^H NMR spectroscopy (formic acid), *n*_electrons_ is electron transfer number (both the production of H_2_ and HCOOH are two-electron processes), *F* is the Faraday constant (96485 C mol^−1^), and *Q* is the total charge passed during the electrolysis of CO_2_. The current density for a given product is calculated by multiplying the total current density with FE of the product. The C_dl_ was determined by measuring CV curves at different scan rates. The potential range for the CV tests was from −0.55 V to −0.50 V vs. Ag/Ag^+^. The EIS spectra were recorded at open circuit potential with an amplitude of 5.0 mV (10^−2^ to 10^5^ Hz), and the value for *R*_ct_ obtained by fitting the EIS spectra using the Zview software (Version 3.1, Scribner Associates, USA)^12,38^.

### EPR analysis

EPR spectra were recorded at room temperature, in continuous-wave mode, on Bruker EMX spectrometers (X-band, *ca*. 9.85 GHz; Q-band, *ca*. 34 GHz), at mw power of ~0.63 mW and modulation amplitude 10 G; spectra reported herein were typically obtained over an average of 20 scans, and a Bruker strong pitch (*g* = 2.0028) reference was used as a calibrator. Theoretical modelling of the spectra was performed with the EasySpin toolbox within Matlab^49^. The intensity of the EPR signal of different samples was normalised to the quantity of sample.

Calculations of the spectra with EasySpin^36^ using the iterative spin Hamiltonian in the Eq. (2)^34,50^:

2 $$\hat H = g\beta B\hat S + D\left[ {\hat S_z^2 - 2/3} \right] + E\left( {\hat S_x^2 + \hat S_y^2} \right)$$

where *D* and *E* are the axial and rhombic zero-field splitting parameters, respectively, to give *g*_xx_ = *g*_yy_ = 2.06–2.07, *g*_zz_ = 2.31–2.36, *D* = 0.33–0.35 cm^−1^ and *E* = 0 (Fig. 2g and Supplementary Figs. 5 and 6 and Supplementary Table 2). These parameters are in excellent agreement with data for binuclear copper acetate^35^ and other Cu(II) systems with a [Cu_2_(OOCR)_4_] paddle wheel structure^36,51^.

The relative content of monomer [uncoupled Cu(II) centre as defect] and binuclear {within [Cu_2_(OOCR)_4_] paddlewheels} centres was calculated from the second integrals from simulated EPR Q-band spectra. At Q-band, the entire *S* = 1 spectrum of the binuclear unit is observed hence defining its relative weighting better than that at X-band where the zero-field splitting is comparable to the microwave frequency. The ratio of monomer to binuclear species is obtained from the weighting of the two spin systems (Supplementary Fig. 7; this is illustrated by the second integrals of the simulated components in Supplementary Fig. 8)^36^. The final monomer to binuclear ratio was obtained by weighting the EPR data by the Boltzmann population of the *S* = 1 state of the binuclear species based upon the Bleaney-Bower equation [(Eq. (3)]^35,36^

3 $$\frac{{nT}}{{nT + nS}} = \frac{{3exp\left( {\frac{{ - {\mathrm{{\Delta}}}E_{ST}}}{{RT}}} \right)}}{{1 + 3exp\left( {\frac{{ - {\mathrm{{\Delta}}}E_{ST}}}{{RT}}} \right)}}$$

where ${\mathrm{{\Delta}}}E_{ST}$ is the singlet (S)-triplet (T) energy gap (*ca*. 300 cm^−1^), *nT* and *nS* are the populations at temperature of *T*, and *R* is the gas constant.

The relative content of free, uncoupled Cu(II) centres as a function of electrolysis time was measured from the second integral of the signal for this species using X-band EPR spectroscopy. EPR spectra of electrodes were collected every 15 min during electrolysis at −1.8 V vs. Ag/Ag^+^. The obtained EPR spectra were transformed to a second integral value, and all data normalised to the surface area of the electrodes.

### DFT calculations

Calculations were based on spin-polarised DFT using projector augmented wave (PAW) methods, as implemented in the Vienna *ab initio* simulation package (VASP)^52^. A plane-wave basis set with a kinetic-energy cut-off of 400 eV was used to expand the wave function of valence electrons. The generalised gradient approximation (GGA) with the Perdew–Burke–Ernzerhof (PBE) functional was used for describing the exchange-correlation interactions^53^. Vacuum space of 20 Å was set to prevent the interaction between two molecules. The Brillouin-zone integration was sampled by single Γ point. The structural relaxations were performed by computing the Hellmann-Feynman forces within the total energy and force convergences of 10^−4^ eV and 0.01 eV/Å, respectively. Based on computational hydrogen electrode (CHE) model^54^, and the *Gibbs* free energy of an adsorbed intermediate from reduced CO_2_ was calculated using ΔG = ΔE + ΔE_ZPE_–TΔS_ads_, where ∆E, ∆E_ZPE_ and T∆S_ads_ are the electronic adsorption energy, zero point energy and entropy corrections, respectively. The corrections of zero point energy and entropy of different species are shown in Supplementary Table 4. Vaspsol, the Poisson-Boltzmann implicit solvation model, was used to describe the effect of solvation^55^, and DFT calculations were implemented via VASP with a dielectric constant of ε = 37.5. The solvation energy was directly contained in the total energy.

## Supplementary information

Supplementary Information

Peer Review File

## Data Availability

All relevant data are available from the authors, and/or are included with the manuscript. All other data supporting the findings of this study are available within the Article and its Supplementary Information, or from the corresponding author upon reasonable request.
