# Supplementary material for: Electro-reduction of carbon dioxide at low over-potential at a metal–organic framework decorated cathode
Source: Nat Commun. 2020 Oct 29;11:5464. doi: 10.1038/s41467-020-19236-4 (PMC7596083; doi:10.1038/s41467-020-19236-4)
Supplement: Supplementary file 1 — Supplementary Information [file 41467_2020_19236_MOESM1_ESM.docx]

**Supplementary Information**

**Electro-reduction of carbon dioxide at low over-potential at a metal-organic framework** **decorated cathode**

Xinchen Kang,^1^ Lili Li,^1^ Alena Sheveleva,^1^ Xue Han,^1^ Jiangnan Li,^1^ Lifei Liu,^2^ Floriana Tuna^1,3^, Eric J. L. McInnes^1^, Buxing Han,^2^* Sihai Yang^1^* and Martin Schröder^1^*

1. Department of Chemistry, University of Manchester, Manchester, M13 9PL (UK)

2. Beijing National Laboratory for Molecular Sciences, CAS Key Laboratory of Colloid, Interface and Chemical Thermodynamics, Institute of Chemistry, Chinese Academy of Sciences, Beijing, 100190 (China)

3. Photon Science Institute, University of Manchester, Manchester M13 9PL (UK)

**Supplementary Figures**


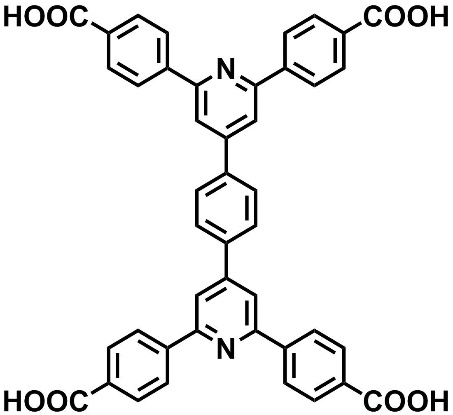


**Supplementary Fig. 1.** View of the structure of H_4_L, 4,4′,4″,4′′′-(1,4-phenylenebis(pyridine-4,2,6-triyl))tetrabenzoic acid.

**
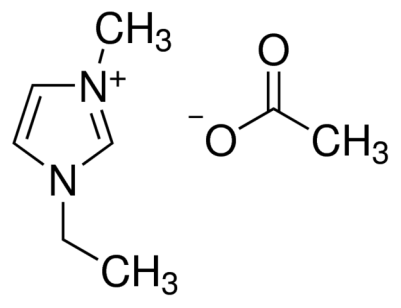
**

**Supplementary Fig. 2.** View of the structure of IL EmimOAc.


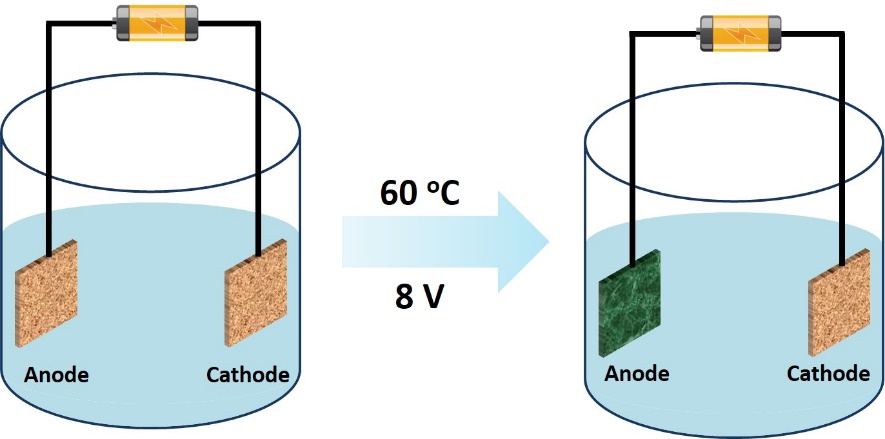


**Supplementary Fig. 3.** Schematic of the synthesis of the Cu_2_(L)-e/Cu electrode.

**
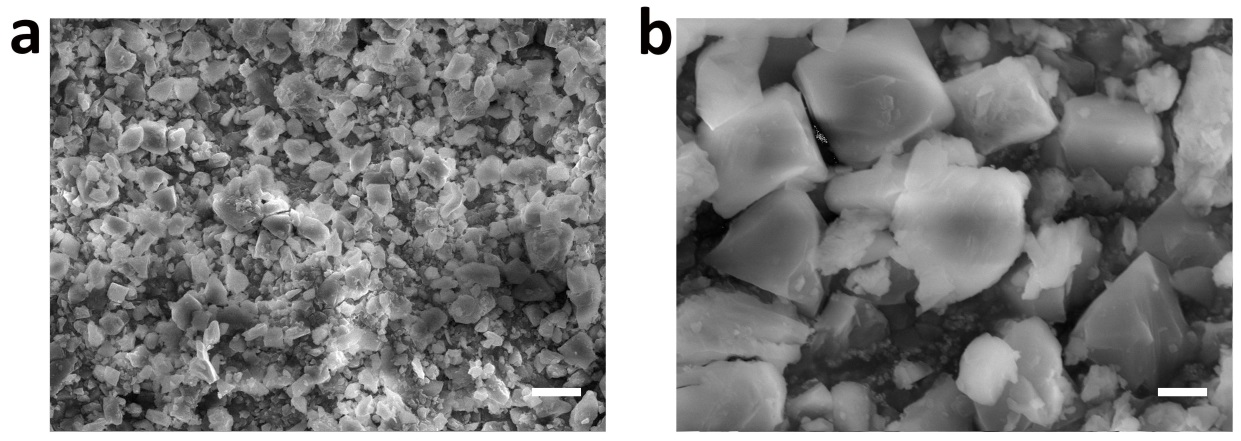
**

**Supplementary Fig. 4.** SEM images of Cu_2_(L)-t. The scale bars in (a) and (b) are 10 µm and 2 µm, respectively.


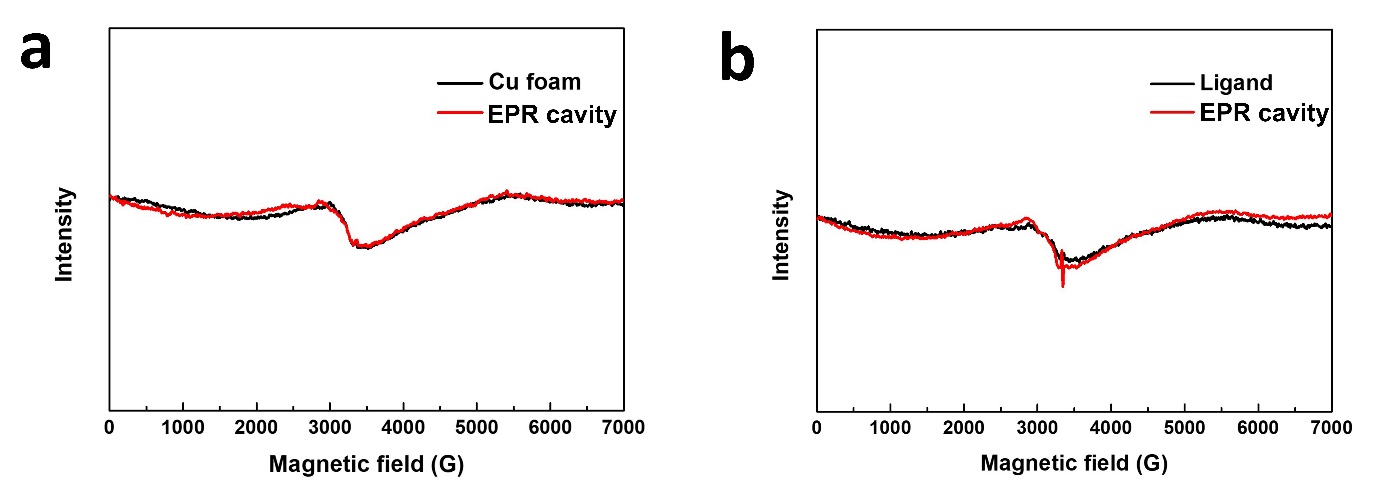


**Supplementary Fig. 5.** X-band EPR spectra of Cu-foam and ligand.


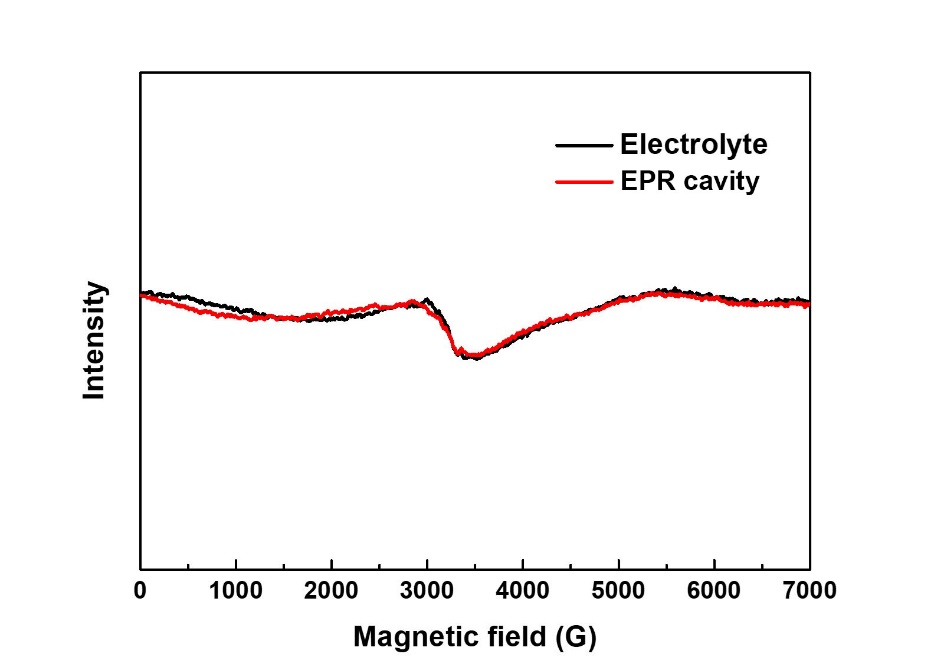


**Supplementary Fig. 6.** X-band EPR spectrum of electrolyte of MOF synthesis.


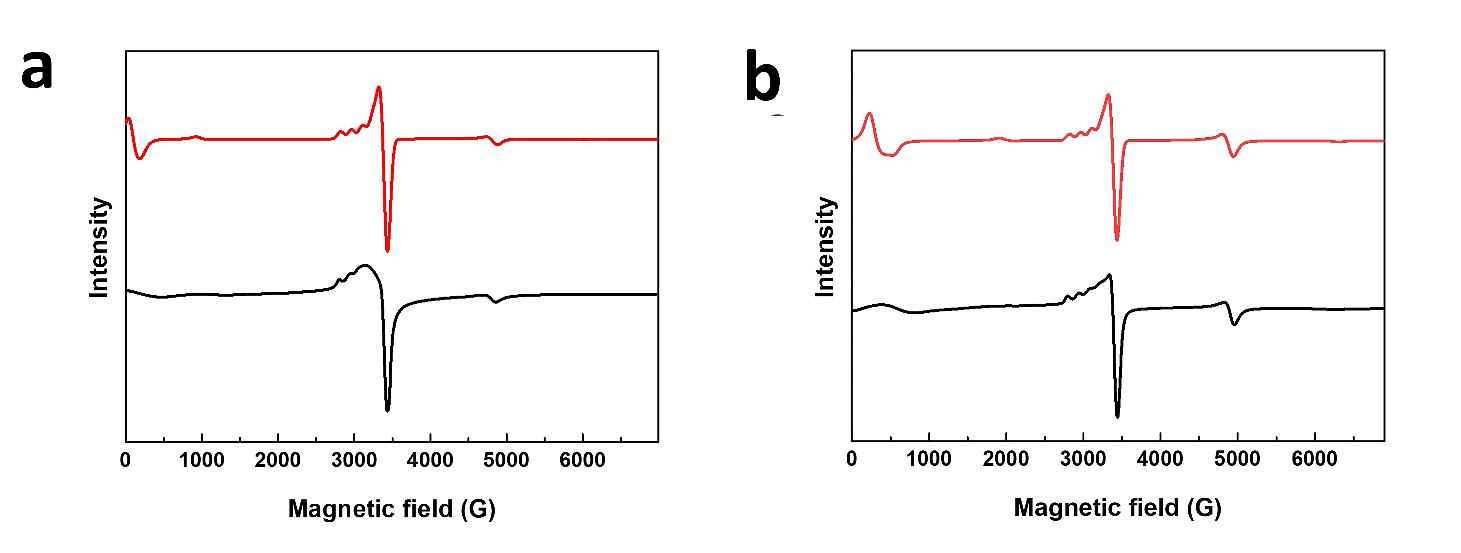


**Supplementary Fig. 7.** X-band EPR spectra of (a) Cu_2_(L)-t and (b) Cu_2_(L)-e at room temperature. Black and red curves refer to experimental data and simulations, respectively. Fitting parameters are given in Supplementary Table 2.


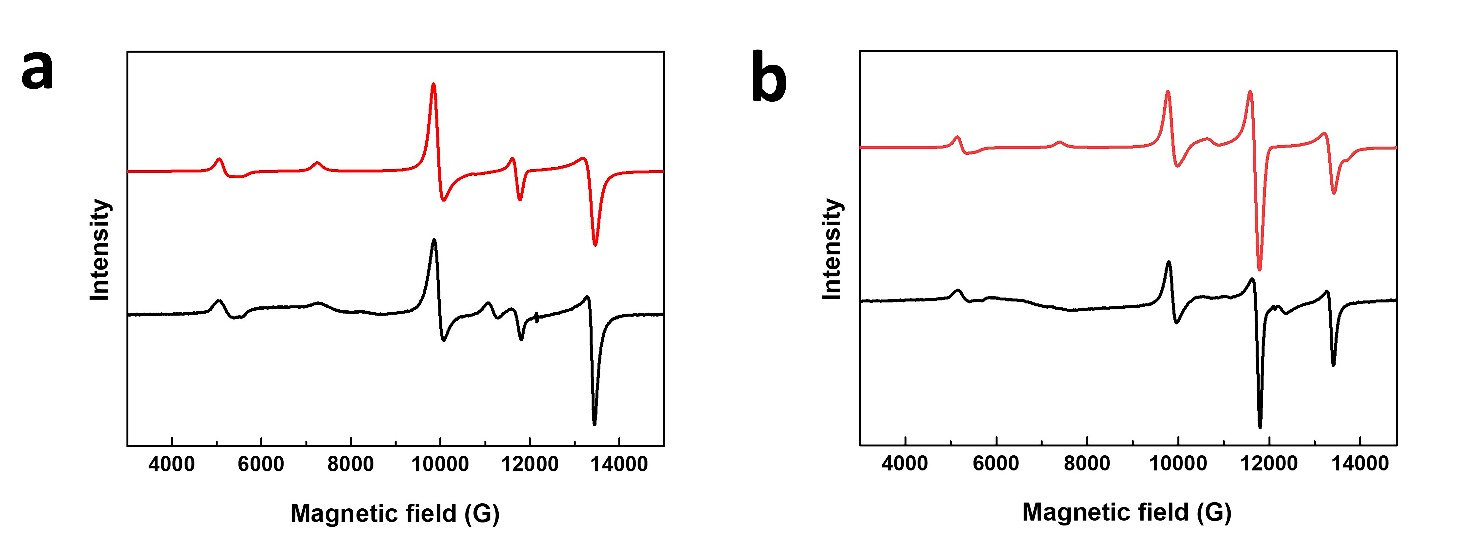


**Supplementary Fig. 8.** Q-band EPR spectra of (a) Cu_2_(L)-t and (b) Cu_2_(L)-e at room temperature. Black and red curves refer to experimental data and simulations, respectively. Fitting parameters are given in Supplementary Table 2.


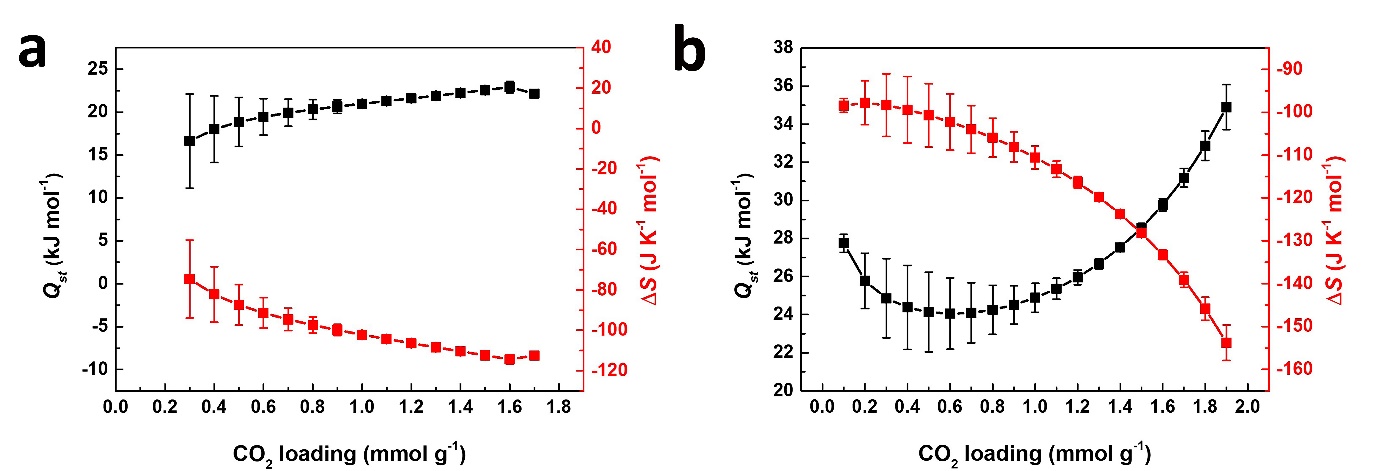


**Supplementary Fig. 9.** Analysis of *Q_st_* and Δ*S* for CO_2_ adsorption in (a) desolvated Cu_2_(L)-t and (b) desolvated Cu_2_(L)-e. Error bars were generated by fitting three adsorption isotherms for CO_2_ at 273 K, 283 K and 298 K to the Van t’ Hoff equation. For Cu_2_(L)-t the error range for *Q_st_*is 0 ~ ±32.9% and for Δ*S*0 ~ ±25.9%. For Cu_2_(L)-e the error range for *Q_st_*is ±0.8% ~ ±9.1% and for Δ*S*±0.6% ~ ±7.8%

**
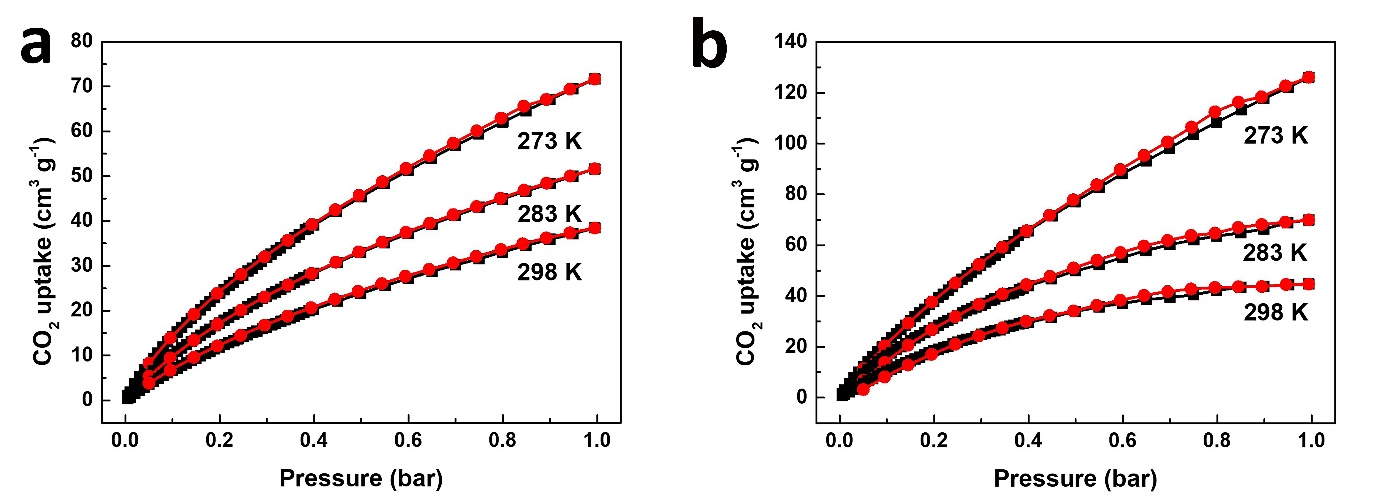
**

**Supplementary Fig. 10.** Adsorption isotherms for CO_2_ in (a) Cu_2_(L)-t and (b) Cu_2_(L)-e at different temperatures.


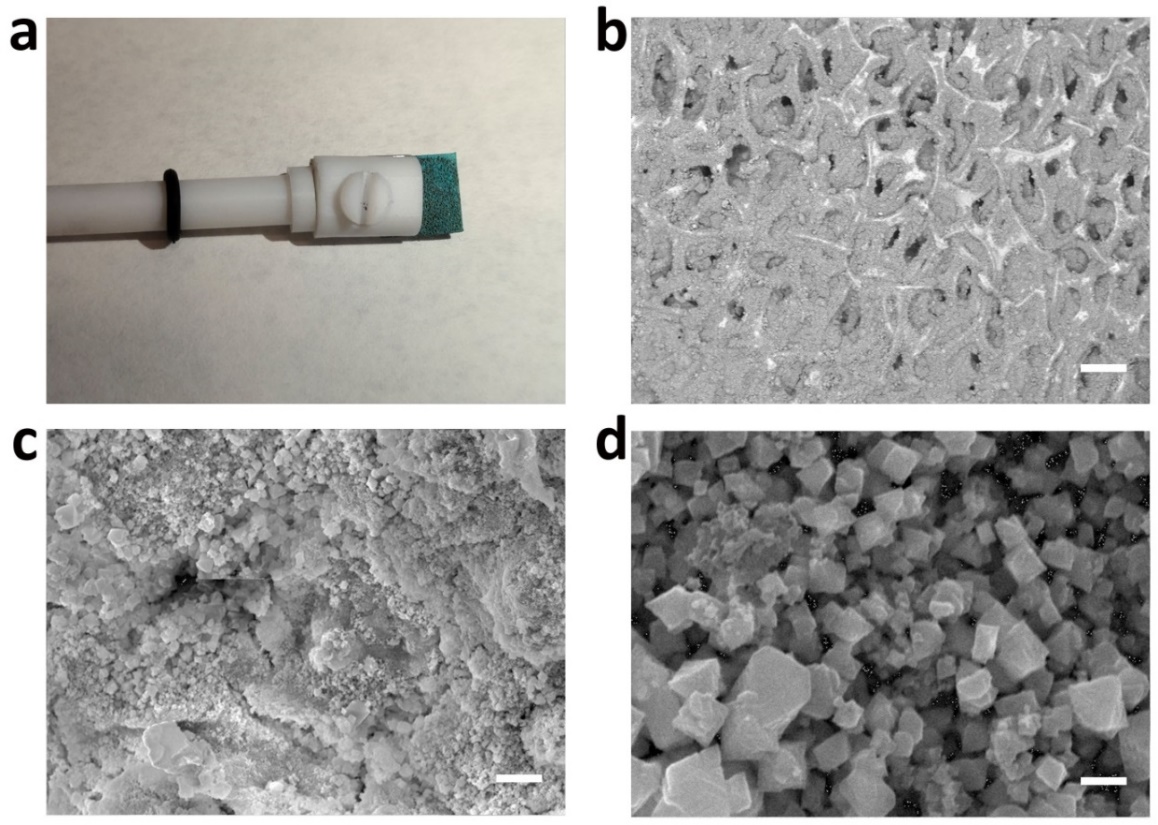


**Supplementary Fig. 11.** (a) View and (b-d) SEM images of HKUST-1-e/Cu (0.5 x 1.0 cm^2^). The scale bars for (b), (c) and (d) are 300 µm, 100 µm and 1 µm, respectively.


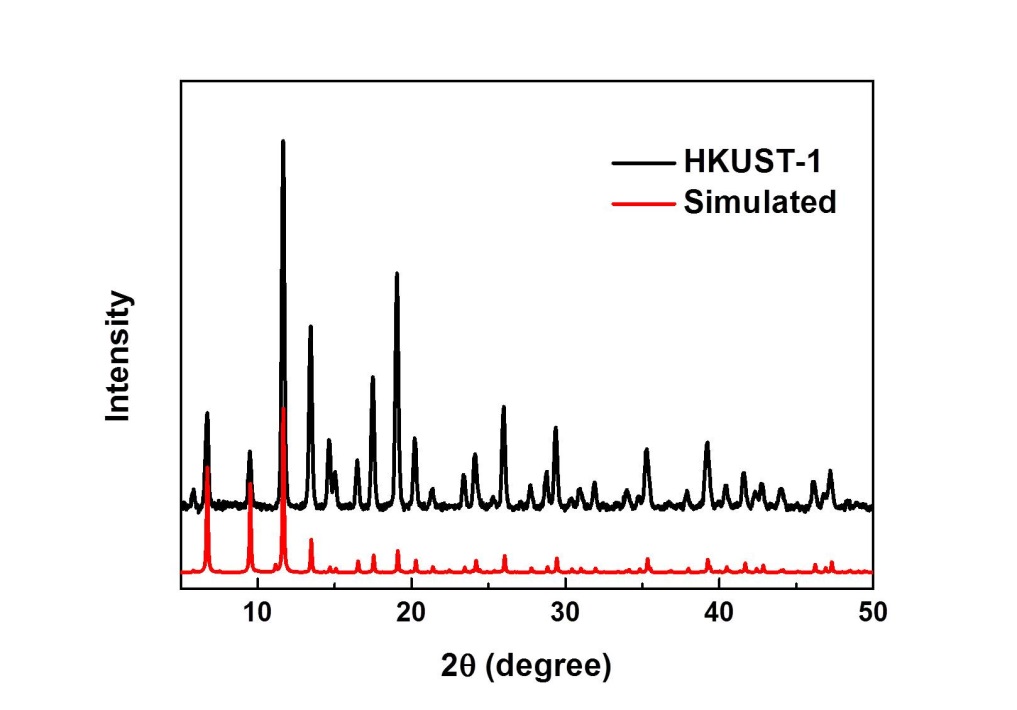


**Supplementary Fig. 12.** View of PXRD patterns for electro-synthesised HKUST-1-e.


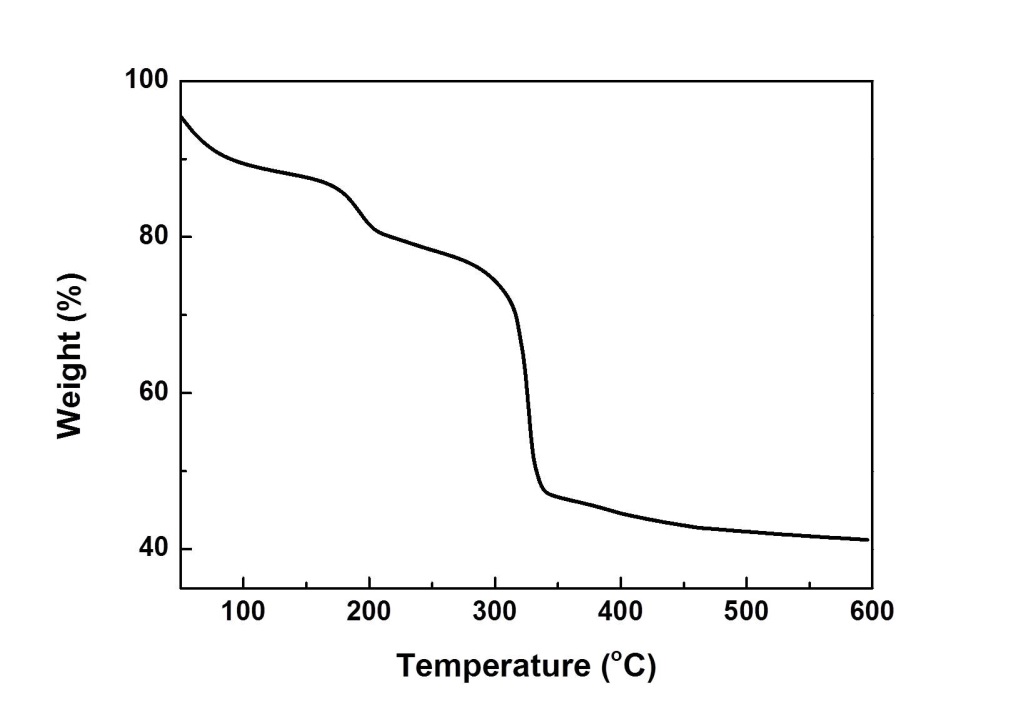


**Supplementary Fig. 13.** TGA curve for HKUST-1-e.


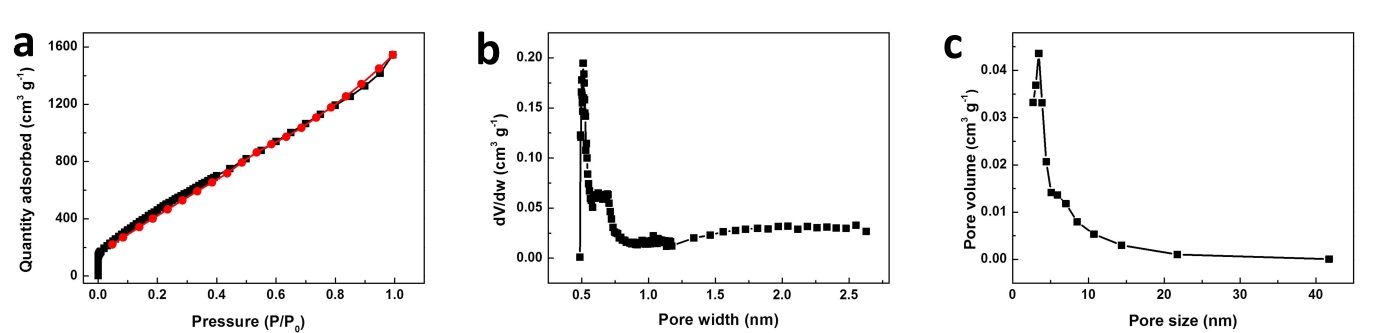


**Supplementary Fig. 14.** BET measurements for HKUST-1-e. (a) N_2_ adsorption/desorption isotherms; (b) micropore size distribution; (c) mesopore size distribution.

**
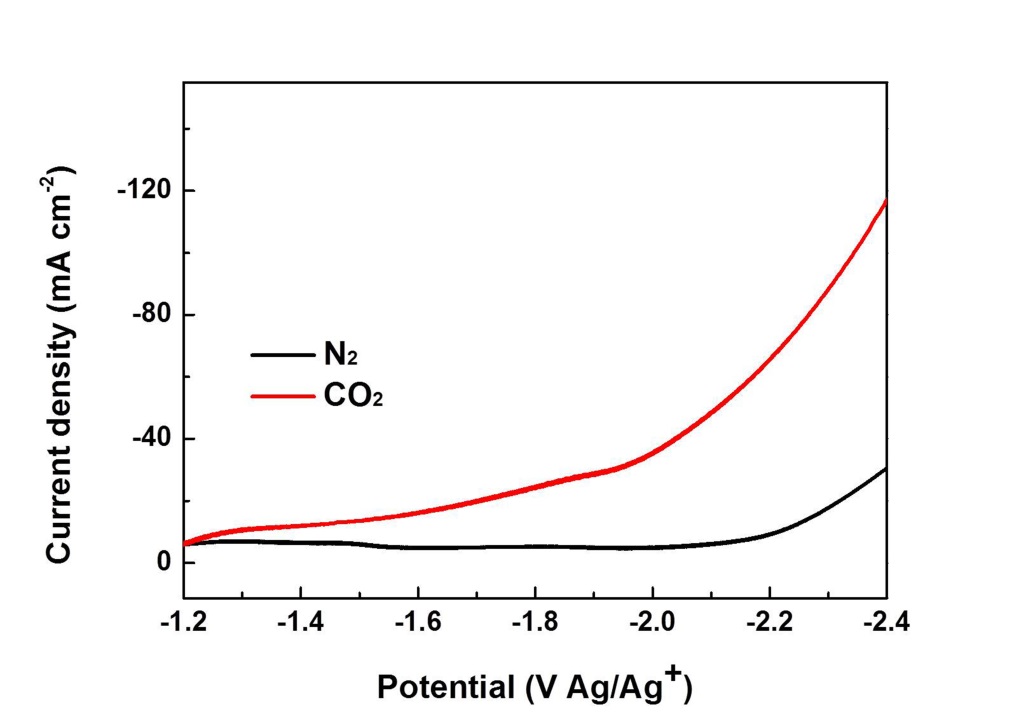
**

**Supplementary Fig. 15.** Linear sweep voltammetry of N_2_- and CO_2_-saturated electrolyte at the Cu_2_(L)-e/Cu electrode.


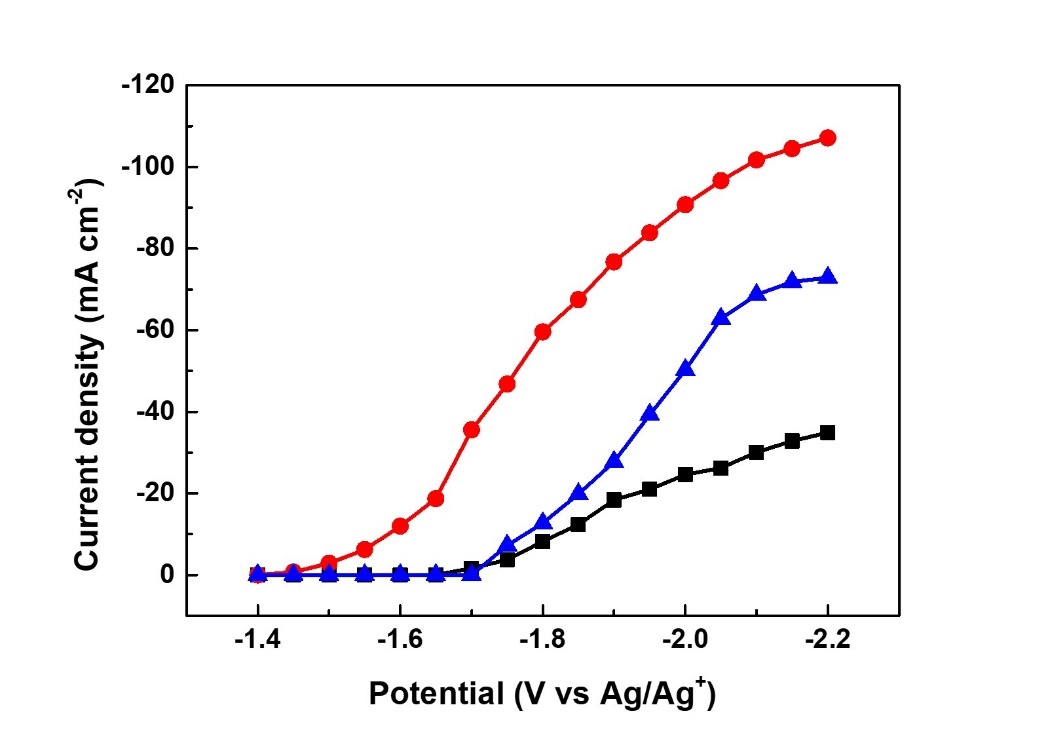


**Supplementary Fig. 16.** Plot of current density of formic acid *vs.* time of CO_2_ electrolysis using Cu_2_(L)-t/CP (black line), Cu_2_(L)-e/Cu (red line) and HKUST-1-e/Cu (blue line) electrodes. The current density of formic acid is calculated by multiplying the total current density by FE_HCOOH_.


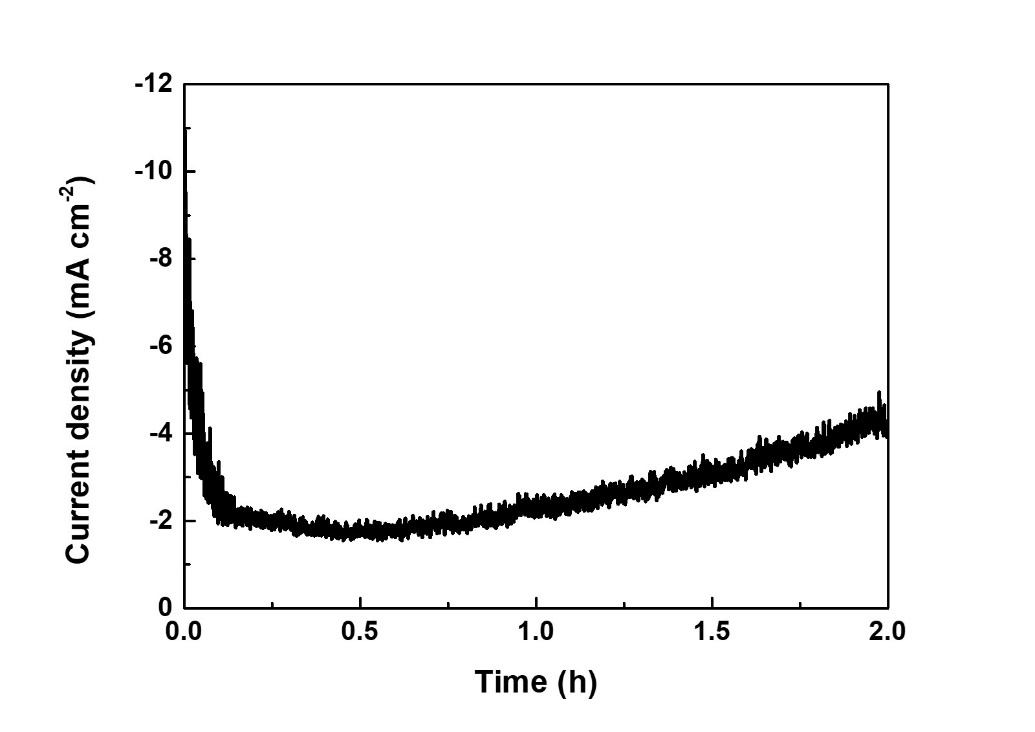


**Supplementary Fig. 17.** Plot of current density *vs.* time for CO_2_ reduction at a free Cu-foam electrode.

**
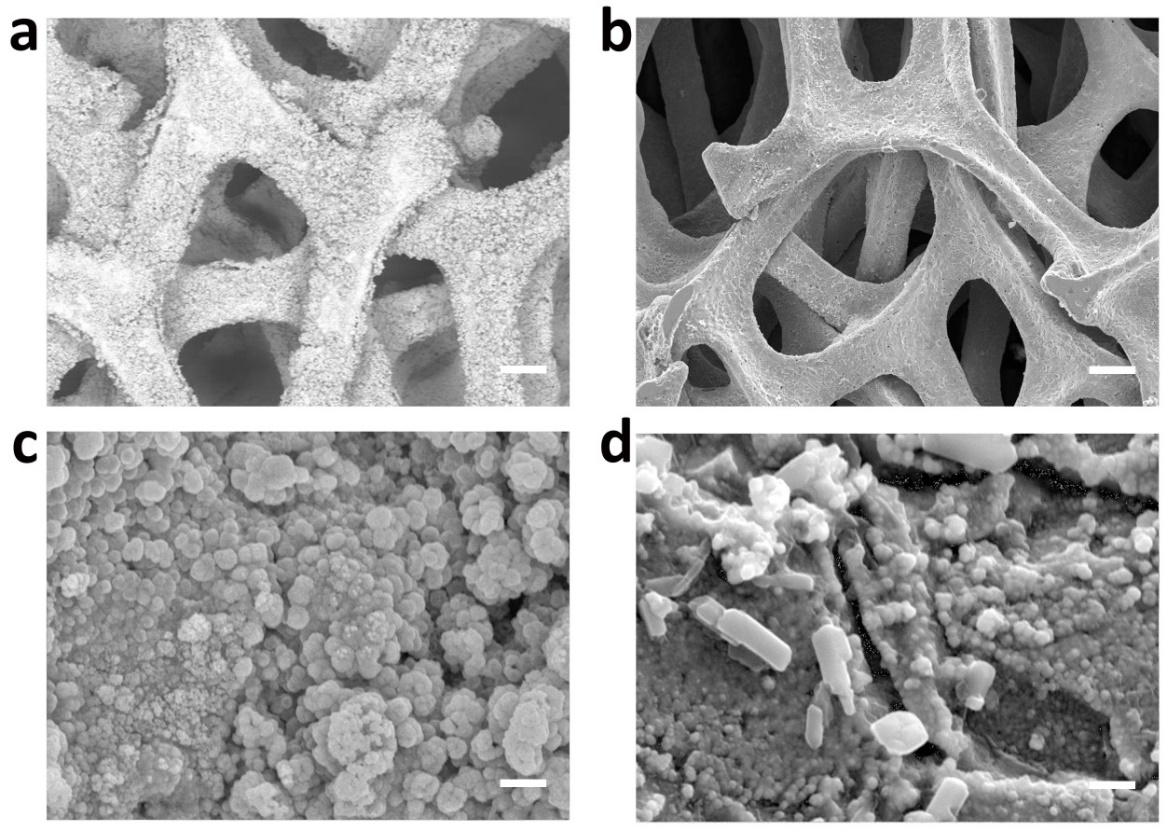
**

**Supplementary Fig. 18.** SEM images of Cu_2_(L)-e/Cu and HKUST-1-e/Cu after electrolysis for 2h. (a, c) Cu_2_(L)-e/Cu; (b, d) HKUST-1-e/Cu. The scale bars are 50 μm, 1 μm, 50 μm and 1 μm for a-d, respectively.

**
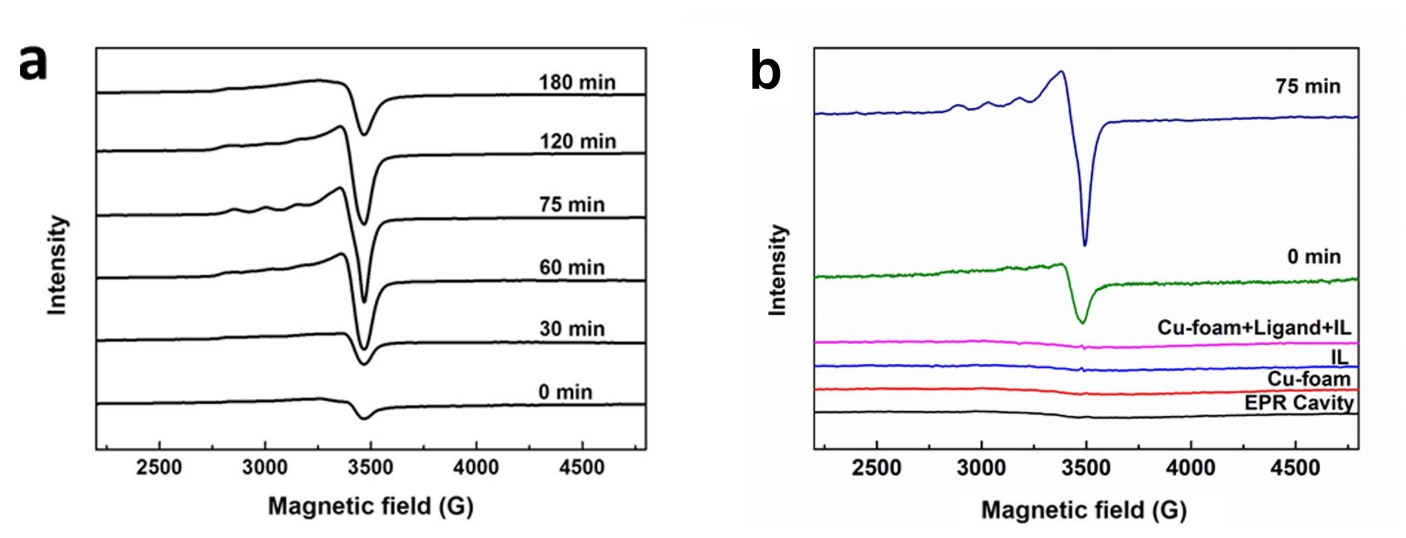
**

**Supplementary Fig. 19.** (a) X-band EPR spectra of the Cu_2_(L)-e/Cu electrode during electrolysis of CO_2_ at −1.8 V *vs* Ag/Ag^+^ as a function of time. (b) Comparisons of the X-band EPR spectra of Cu_2_(L)-e/Cu electrode and other related components. The electrodes were taken out of the electrochemical cell and EPR spectra of the electrodes were collected every 15 minutes during electrolysis at −1.8 V vs. Ag/Ag+, taking care to maintain the experimental conditions unchanged


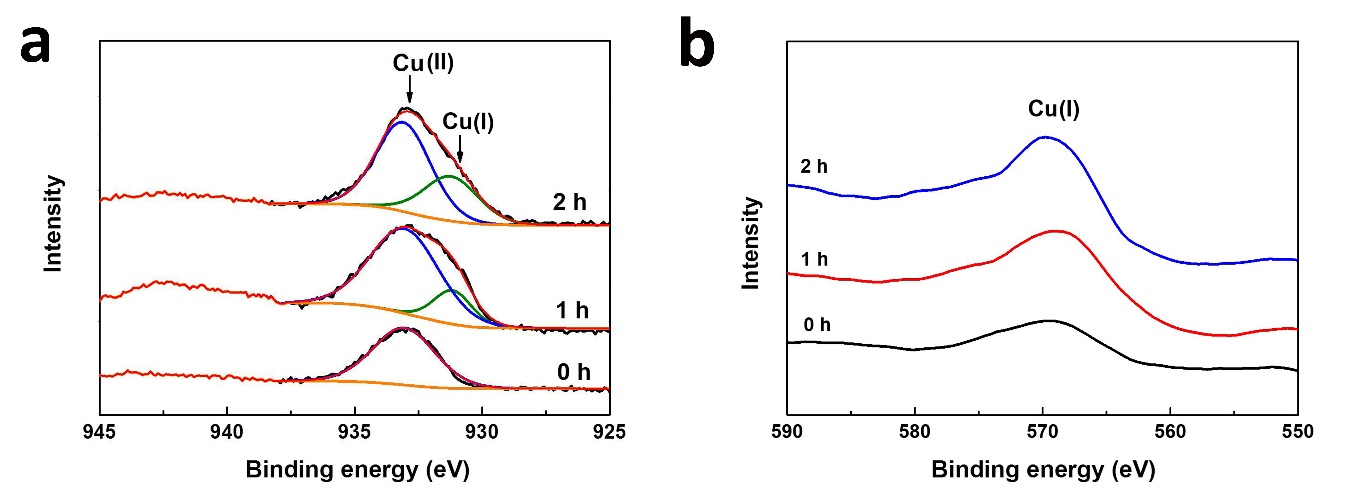


**Supplementary Fig. 20.** Dependence of (a) XPS and (b) Auger spectra of Cu_2_(L)-e/Cu on time of electrolysis.

**
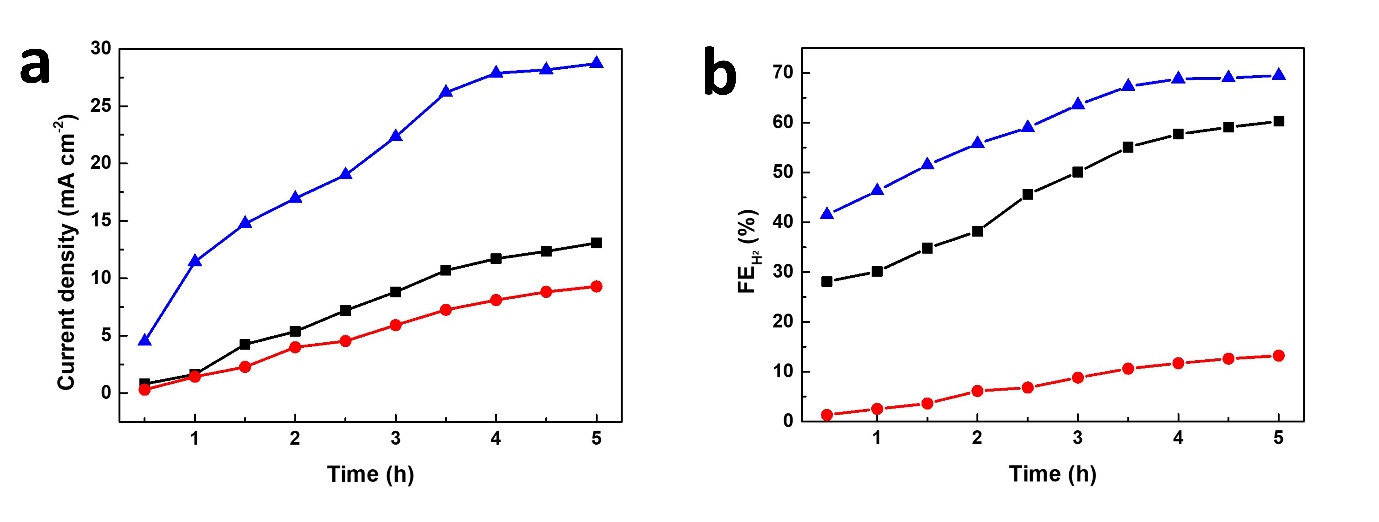
**

**Supplementary Fig. 21.** Plots of current density of (a) H_2_ and (b) FE_H2_ *vs.* time during electrolysis of CO_2_ using Cu_2_(L)-t/CP (black line), Cu_2_(L)-e/Cu (red line) and HKUST-1-e/Cu (blue line) at −1.8 V *vs* Ag/Ag^+^. The current density of H_2_ is calculated by multiplying the total current density by FE_H2_.


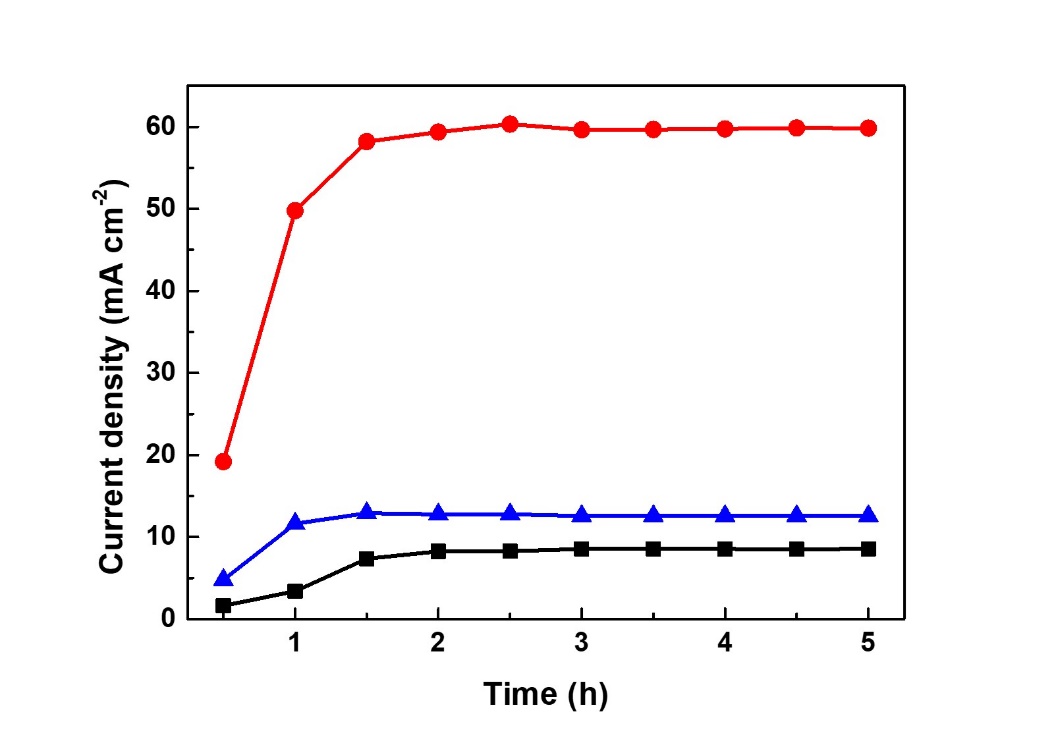


**Supplementary Fig. 22.** Plot of the current density of formic acid *vs.* time during electrolysis of CO_2_ over Cu_2_(L)-t/CP (black line), Cu_2_(L)-e/Cu (red line) and HKUST-1-e/Cu (blue line) at −1.8 V *vs* Ag/Ag^+^. The current density of formic acid is calculated by multiplying the total current density by FE_HCOOH_.

**
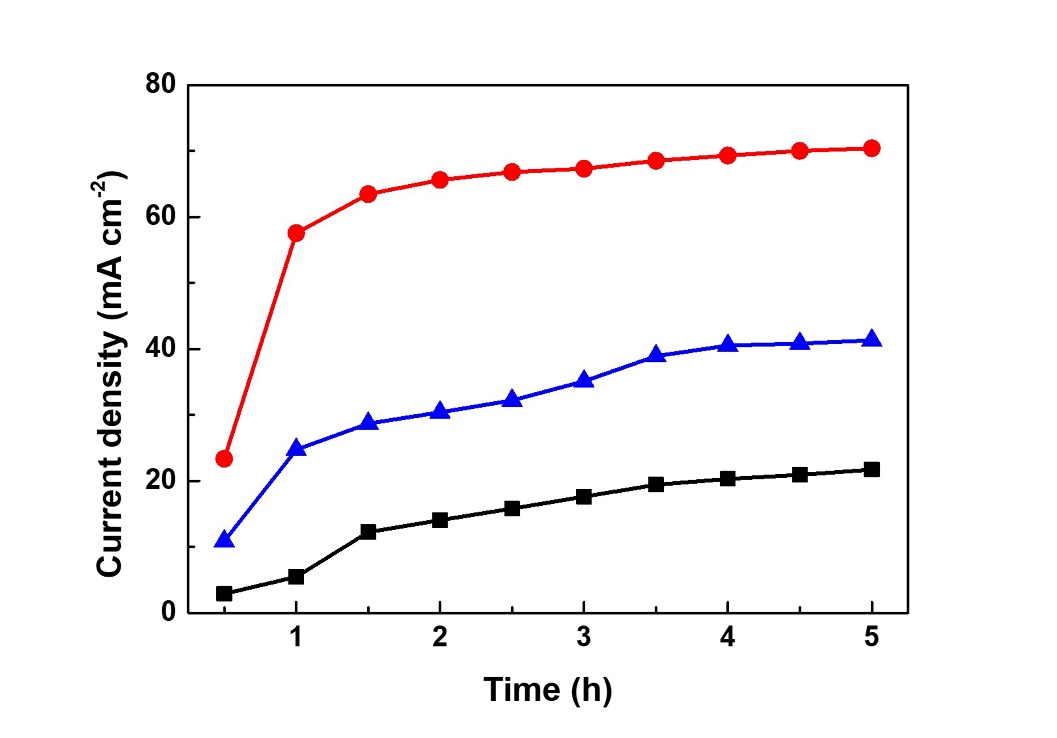
**

**Supplementary Fig. 23.** Plots of total current density *vs.* time during electrolysis of CO_2_ using Cu_2_(L)-t/CP (black line), Cu_2_(L)-e/Cu (red line) and HKUST-1-e/Cu (blue line) at −1.8 V *vs* Ag/Ag^+^.


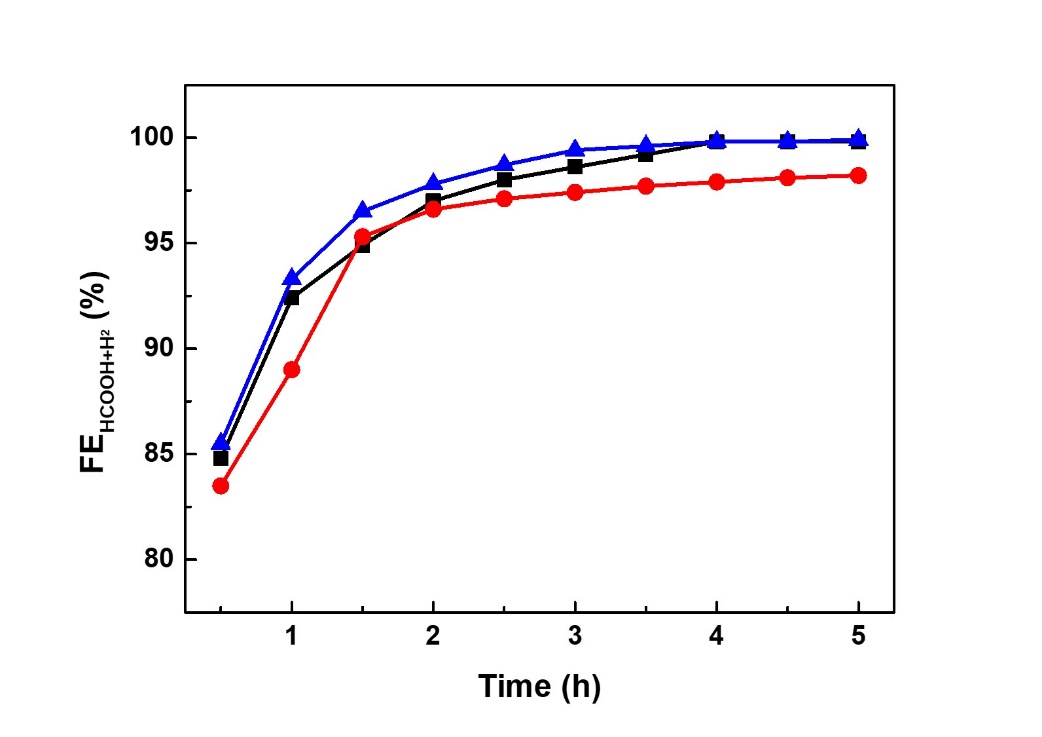


**Supplementary Fig. 24.** Plot of FE_HCOOH+H2_ *vs.* time for electrolysis of CO_2_ using Cu_2_(L)-t/CP (black line), Cu_2_(L)-e/Cu (red line) and HKUST-1-e/Cu (blue line) at −1.8 V *vs* Ag/Ag^+^.


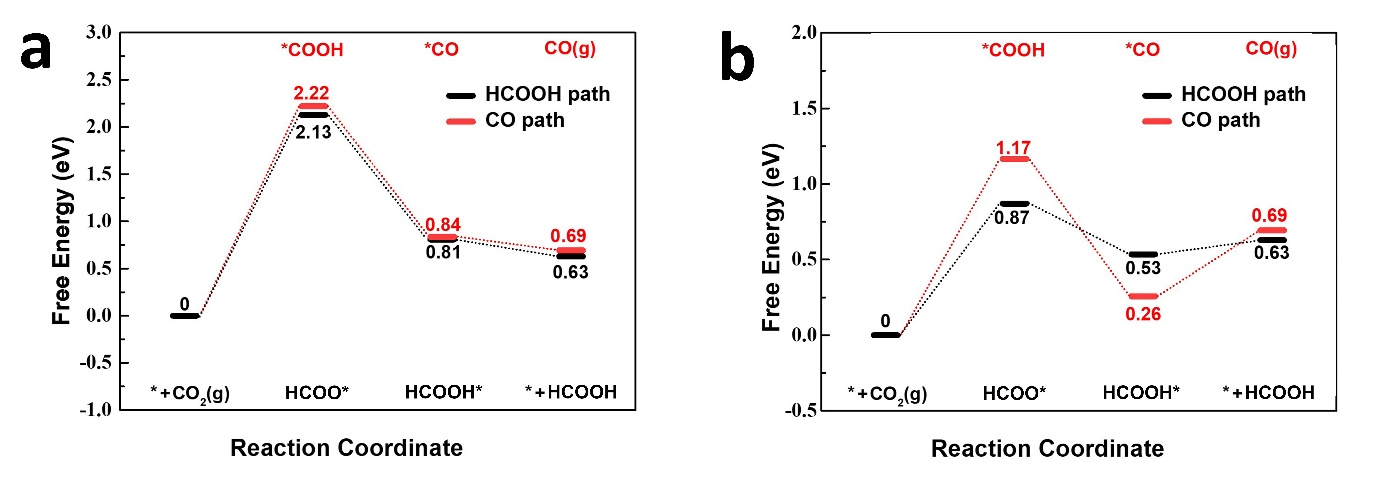


**Supplementary Fig. 25.** DFT calculated diagram of *Gibbs* free energy of the electro-reduction of CO_2_ over (a) pristine and (b) defect Cu_2_(L) over different pathways.


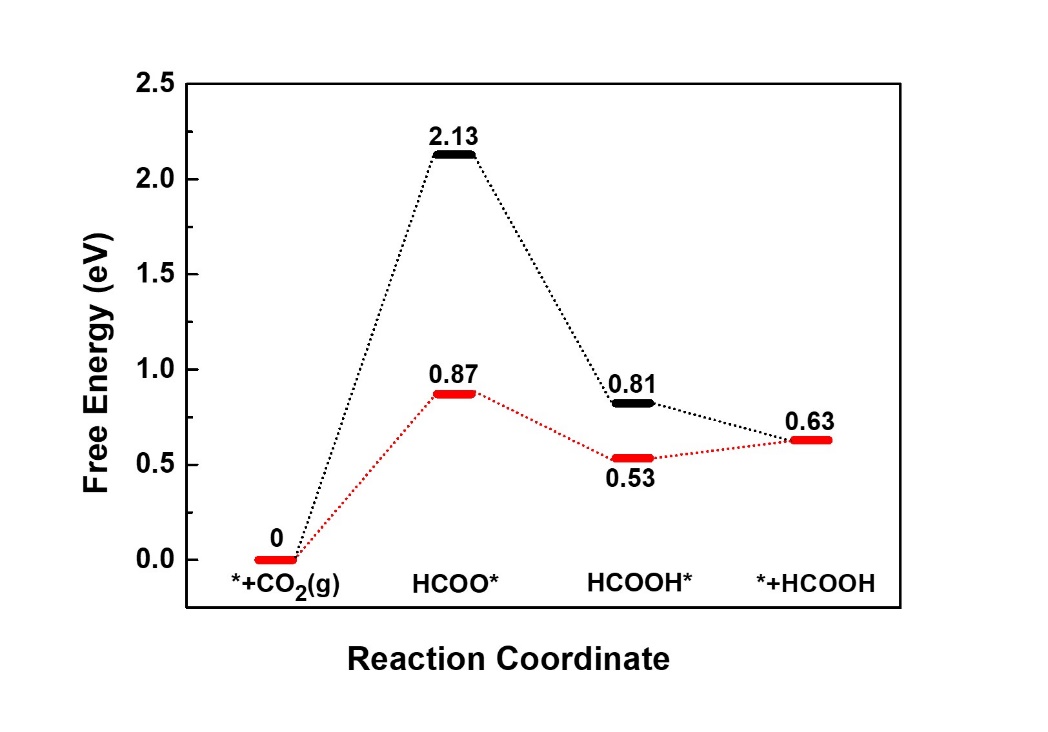


**Supplementary Fig. 26.** DFT calculated diagram of *Gibbs* free energy of electro-reduction of CO_2_ to formic acid over pristine (black lines) and defect Cu_2_(L) (red lines).


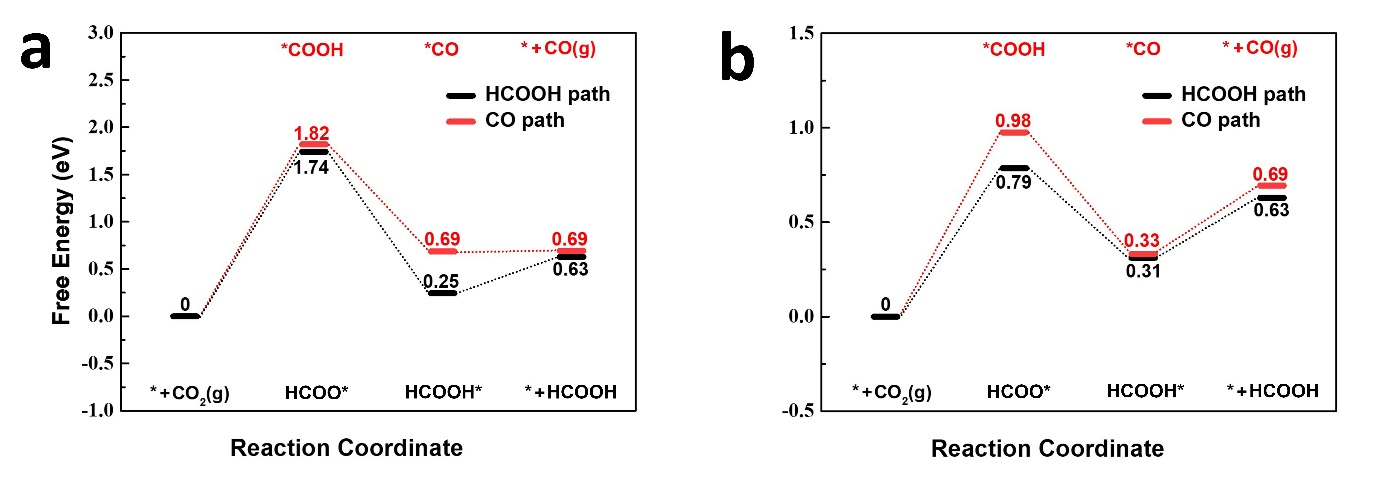


**Supplementary Fig. 27.** DFT calculated diagram of *Gibbs* free energy of the electro-reduction of CO_2_ over (a) pristine and (b) defect Cu_2_(L) over different pathways including solvation effects.


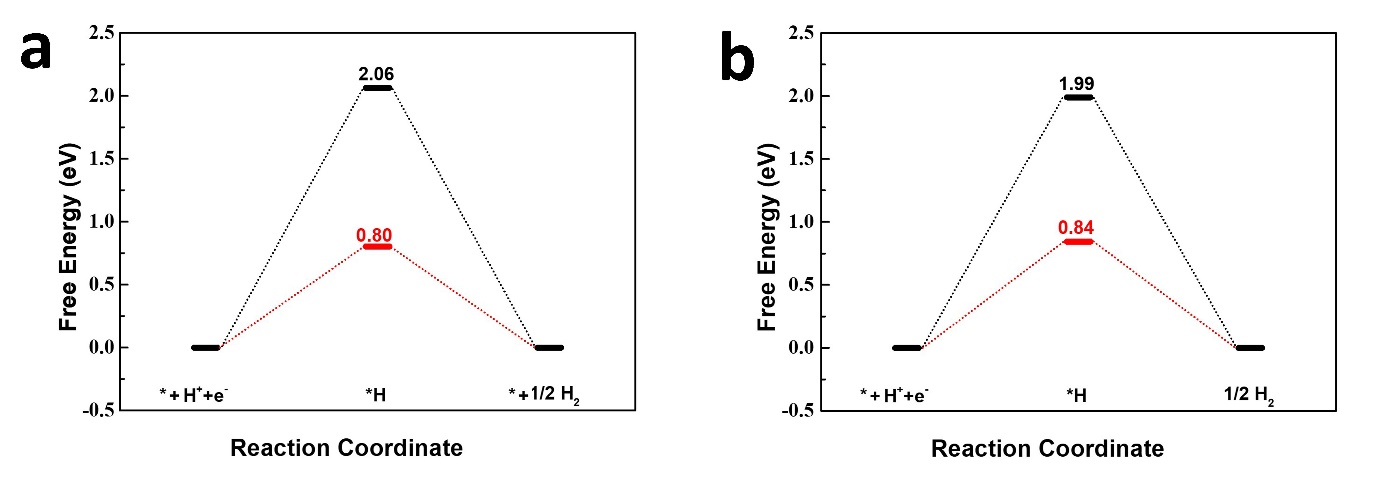


**Supplementary Fig. 28.** DFT calculation of *Gibbs* free energy for H_2_ evolution reaction over pristine (black lines) and defect Cu_2_(L) (red lines) (a) before and (b) after inclusion of solvation effects.

**Supplementary Tables**

**Supplementary Table 1.** Summary of the porosity, BET surface area (*S*), average mesopore size (*D*) and total mesopore volume (*V*) for materials in this study.

| Samples | *S*_total_  (m^2^ g^-1^) | *S*_meso_  (m^2^ g^-1^) | *D*_meso_  (nm) | *V*_meso_  (cm^3^ g^-1^) | Cu:L^†^ (mol:mol) |
| --- | --- | --- | --- | --- | --- |
| Cu_2_(L)-t | 1950 | 369 | 3.5 | 0.32 | 2.0 |
| Cu_2_(L)-e | 1449 | 1363 | 4.8 | 1.89 | 2.4 |
| HKUST-1-e^‡^ | 2042 | 1976 | 4.6 | 2.36 | 1.6 |

*S*_total_ = total surface area; *S*_meso_ = BJH desorption cumulative surface area of pores; *D*_meso_ = mesopore size; *V*_meso_ = mesopore volume.

^†^The Cu:L ratio was calculated from ICP and elemental analysis results.

^‡^HKUST-1-e was prepared using the same method of electro-synthesis as for Cu_2_(L)-e.

**Supplementary Table 2.** Data and simulation parameters for Cu_2_(L)-e and Cu_2_(L)-t derived from CW EPR spectra at X-band and Q-band.^*^

| Species | Parameter | Cu_2_(L)-e | | Cu_2_(L)-t | |
| --- | --- | --- | --- | --- | --- |
|  |  | X-band | Q-band | X-band | Q-band |
| binuclear paddlewheel  [Cu_2_(OOCR)_4_] | *g*-factor | 2.07  2.31 | 2.065  2.32 | 2.06  2.36 | 2.062  2.36 |
|  | *D*/cm^-1^ | 0.34 | 0.355 | 0.335 | 0.335 |
|  | *E* | 0 | 0 | 0 | 0 |
|  | lw/G | 5  20 | 10  10 | 12  20 | 10  10 |
| monomer  free Cu(II) | *g*-factor | 2.07  2.32 | 2.075  2.32 | 2.07  2.32 | 2.075  2.32 |
|  | *A*/MHz | 33.6  450 | 33.6  450 | 33.6  450 | 33.6  450 |
|  | lw/G | 20.6 | 10 | 17 | 10 |

^*^lw is the homogeneous Lorentzian linewidth; *g* and *A* are the *g-*value and Cu hyperfine coupling constants, respectively; the two values represent the parallel and perpendicular components; *D* and *E* are the axial and rhombic zero-field splitting parameters of the *S* = 1 state, respectively

**Supplementary Table 3.** The catalytic performance for electrochemical reduction of CO_2_ to formic acid using various electrodes in organic electrolyte.

| Entry | electrode | electrolyte | P_1_ (V) | P_2_ (V) | *j*  (mA·cm^-2^)^†^ | FE_HCOOH_ (%)^‡^ | Ref. |
| --- | --- | --- | --- | --- | --- | --- | --- |
| 1 | Pb | BmimPF_6_/MeCN/H_2_O (30/65/5) | −1.99^║^ | −2.2^║^ | 17.8 | 95.3 | 1 |
| 2 | Sn | BmimPF_6_/MeCN/H_2_O (30/65/5) | −2.0^║^ | −2.2^║^ | 15.8 | 95.0 | 1 |
| 3 | MoP@In-PC | BmimPF_6_/MeCN/H_2_O (30/65/5) | −1.90^║^ | −2.2^║^ | 43.8 | 96.5 | 2 |
| 4 | Pb-PhyA | BzmimBF_4_ /MeCN/H_2_O (12.8/77.3/9.9) | −2.05^║^ | −2.25^║^ | 30.5 | 92.7 | 3 |
| 5 | SnO_2_@N-PC | 0.5 M BmimPF_6_/MeCN | −1.9^║^ | −2.2^║^ | 28.4 | 94.1 | 4 |
| 6 | Cu_2_(CuTCPP) | 0.5 M EmimBF_4_/MeCN/H_2_O (1 M) | −1.2^║^ | −1.55^║^ | 3.5 | 68.4 | 5 |
| 7 | Sn powder | 0.5 M EmimN(CN)_2_/H_2_O | −0.4^┴^ | −1.2^┴^ | 0.633 | 81.9 | 6 |
| 8 | Bi | 250 mM BmimPF_6_/MeCN/0.1 M TBAPF_6_ | −1.6^┬^ | −1.8^┬^ | 27 | 77 | 7 |
| 9 | Cu_2_(L)-t/CP | 0.5 M EmimBF_4_/MeCN | −1.7^║^ | −1.9^║^ | 24.0 | 76.6 | This work |
| 10 | Cu_2_(L)-e/Cu | 0.5 M EmimBF_4_/MeCN | −1.45^║^ | −1.8^║^ | 65.8 | 90.5 | This work |
| 11 | HKUST-1-e/Cu | 0.5 M EmimBF_4_/MeCN | −1.75^║^ | −2.05^║^ | 107 | 62.2 | This work |

P_1_ = Onset potential for formic acid.

P_2_ = Potential at highest value of FE_HCOOH_.

^†^ Current density at P_2_.

^‡^ FE_HCOOH_ at P_2_.

^║^vs Ag/Ag^+^.

^┴^vs RHE.

^┬^vs SCE.

**Supplementary Table 4.** Correction values of zero-point energy (ZPE) and entropy of the adsorbed species.

| electrode | Intermediates | ZPE (eV) | TS (eV) |
| --- | --- | --- | --- |
| Pristine Cu_2_(L) | *COOH | 0.593 | 0.129 |
|  | *CO | 0.182 | 0.179 |
|  | HCOO* | 0.570 | 0.163 |
|  | HCOOH* | 0.909 | 0.139 |
| Defect Cu_2_(L) | *COOH | 0.605 | 0.117 |
|  | *CO | 0.194 | 0.100 |
|  | HCOO* | 0.583 | 0.108 |
|  | HCOOH* | 0.932 | 0.232 |

*refers to the binding site of an intermediate or product to Cu within the MOF.

**Structural models and atomic coordinates of all intermediates for DFT studies**

In all these studies * refers to the binding site of an intermediate or product to Cu within the MOF.

**Pristine Cu_2_(L)*COOH**

**
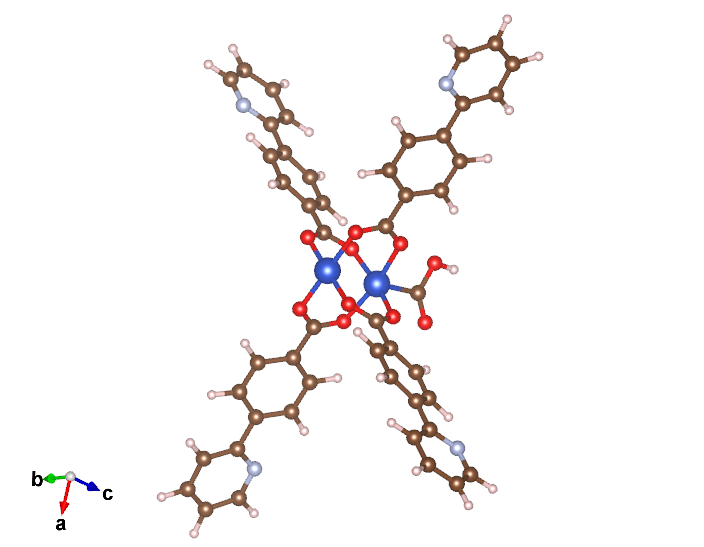
**

The lattice vector (Å) of the structural model.

| a | 30 | 0 | 0 |
| --- | --- | --- | --- |
| b | 0 | 30 | 0 |
| c | 0 | 0 | 30 |

**Supplementary Table 5.** Atomic coordinates for pristine Cu_2_(L)*COOH

| O | 0.550841053 | 0.56073929 | 0.479623467 |
| --- | --- | --- | --- |
| O | 0.540100415 | 0.561694 | 0.554863676 |
| O | 0.447247419 | 0.56152583 | 0.541130161 |
| O | 0.459120529 | 0.56041266 | 0.466030423 |
| O | 0.447648893 | 0.467960971 | 0.540464386 |
| O | 0.459423758 | 0.469051619 | 0.465370846 |
| O | 0.55092469 | 0.468877499 | 0.479848205 |
| O | 0.540582048 | 0.468510514 | 0.555188026 |
| O | 0.509636663 | 0.520169275 | 0.650846705 |
| O | 0.436992425 | 0.506267913 | 0.633595135 |
| C | 0.559613267 | 0.574208937 | 0.519229014 |
| C | 0.596602619 | 0.607052738 | 0.524586522 |
| C | 0.615237495 | 0.628568711 | 0.487538957 |
| C | 0.650309796 | 0.658351274 | 0.493180652 |
| C | 0.668115988 | 0.666963967 | 0.535712953 |
| C | 0.648942764 | 0.645364728 | 0.57263236 |
| C | 0.613552208 | 0.616013798 | 0.567245761 |
| C | 0.706373406 | 0.697549325 | 0.542565697 |
| C | 0.751313788 | 0.73449937 | 0.593064858 |
| C | 0.777459148 | 0.753473914 | 0.559538224 |
| C | 0.766926172 | 0.743259036 | 0.515441753 |
| C | 0.731145913 | 0.715033734 | 0.506795326 |
| C | 0.439121969 | 0.573930706 | 0.50127352 |
| C | 0.402300975 | 0.606834169 | 0.495356183 |
| C | 0.383525998 | 0.627546563 | 0.53280079 |
| C | 0.348245424 | 0.657117605 | 0.527895677 |
| C | 0.330321083 | 0.666489599 | 0.485591611 |
| C | 0.349712946 | 0.645831253 | 0.448205665 |
| C | 0.385331788 | 0.616559787 | 0.452907217 |
| C | 0.291855879 | 0.696997571 | 0.479542572 |
| C | 0.246322619 | 0.734389176 | 0.429862348 |
| C | 0.220597485 | 0.753124108 | 0.463836987 |
| C | 0.231695053 | 0.742603889 | 0.507733679 |
| C | 0.267505522 | 0.714214156 | 0.515759509 |
| C | 0.439295214 | 0.455733175 | 0.50062022 |
| C | 0.402078646 | 0.423227738 | 0.494717605 |
| C | 0.383632101 | 0.402169722 | 0.532148902 |
| C | 0.348266645 | 0.372718682 | 0.527226772 |
| C | 0.329892209 | 0.36389412 | 0.484996979 |
| C | 0.348856989 | 0.384998767 | 0.447668814 |
| C | 0.384570652 | 0.414138908 | 0.452359096 |
| C | 0.291259555 | 0.333591628 | 0.478996978 |
| C | 0.245687334 | 0.296060574 | 0.429500285 |
| C | 0.219597419 | 0.27793126 | 0.463545476 |
| C | 0.230555382 | 0.28883567 | 0.50737684 |
| C | 0.266572332 | 0.316986707 | 0.515244516 |
| C | 0.560020631 | 0.455916603 | 0.519536773 |
| C | 0.597285604 | 0.423411718 | 0.524845176 |
| C | 0.615710308 | 0.401856434 | 0.487741307 |
| C | 0.650875406 | 0.37215368 | 0.493218116 |
| C | 0.668957925 | 0.363668338 | 0.535649441 |
| C | 0.650021291 | 0.385411796 | 0.572632901 |
| C | 0.61458954 | 0.414721072 | 0.56740817 |
| C | 0.707278092 | 0.333125812 | 0.542390407 |
| C | 0.75219082 | 0.295809777 | 0.592661552 |
| C | 0.778379436 | 0.277056791 | 0.559040117 |
| C | 0.7678954 | 0.287615018 | 0.515018648 |
| C | 0.732185745 | 0.315998302 | 0.506555538 |
| C | 0.480176233 | 0.51408341 | 0.62402028 |
| H | 0.601829601 | 0.621703639 | 0.454464178 |
| H | 0.663736001 | 0.675078364 | 0.463830581 |
| H | 0.662598397 | 0.652218044 | 0.605586752 |
| H | 0.598755198 | 0.599236334 | 0.595883269 |
| H | 0.805289096 | 0.775332757 | 0.567898732 |
| H | 0.722928266 | 0.706352203 | 0.472499271 |
| H | 0.397055158 | 0.620036921 | 0.565669631 |
| H | 0.334772666 | 0.673139222 | 0.557608225 |
| H | 0.335931955 | 0.653276025 | 0.41542129 |
| H | 0.400096699 | 0.600468041 | 0.423855679 |
| H | 0.192727748 | 0.775111483 | 0.455969418 |
| H | 0.276191321 | 0.705391077 | 0.549907395 |
| H | 0.397478204 | 0.409371838 | 0.564952497 |
| H | 0.33502752 | 0.356383395 | 0.556872251 |
| H | 0.334659859 | 0.37806464 | 0.414948634 |
| H | 0.399034549 | 0.430606691 | 0.423363401 |
| H | 0.191606488 | 0.256072182 | 0.455762763 |
| H | 0.275095857 | 0.32621716 | 0.549325883 |
| H | 0.602035147 | 0.408579073 | 0.454756584 |
| H | 0.66409496 | 0.355351193 | 0.463821806 |
| H | 0.663987327 | 0.378719965 | 0.605494047 |
| H | 0.600045301 | 0.431669132 | 0.596073312 |
| H | 0.806142013 | 0.255071843 | 0.567302264 |
| H | 0.724069579 | 0.324978629 | 0.472304051 |
| H | 0.758544405 | 0.741345453 | 0.628182608 |
| H | 0.78648103 | 0.756955977 | 0.487969147 |
| H | 0.238806259 | 0.741591377 | 0.394873262 |
| H | 0.212567313 | 0.756219622 | 0.535533668 |
| H | 0.238252317 | 0.288569115 | 0.394560498 |
| H | 0.211178957 | 0.275682634 | 0.535232339 |
| H | 0.759319473 | 0.288657586 | 0.627738503 |
| H | 0.787444869 | 0.27406466 | 0.487474853 |
| H | 0.433805833 | 0.506299104 | 0.666366103 |
| N | 0.716817623 | 0.707426021 | 0.58525142 |
| N | 0.280909634 | 0.707248494 | 0.437073718 |
| N | 0.280470191 | 0.322982432 | 0.436574682 |
| N | 0.71765084 | 0.322896963 | 0.585018239 |
| Cu | 0.492726228 | 0.514849645 | 0.555614087 |
| Cu | 0.505258186 | 0.514767856 | 0.47201512 |

**Pristine Cu_2_(L)*CO**

**
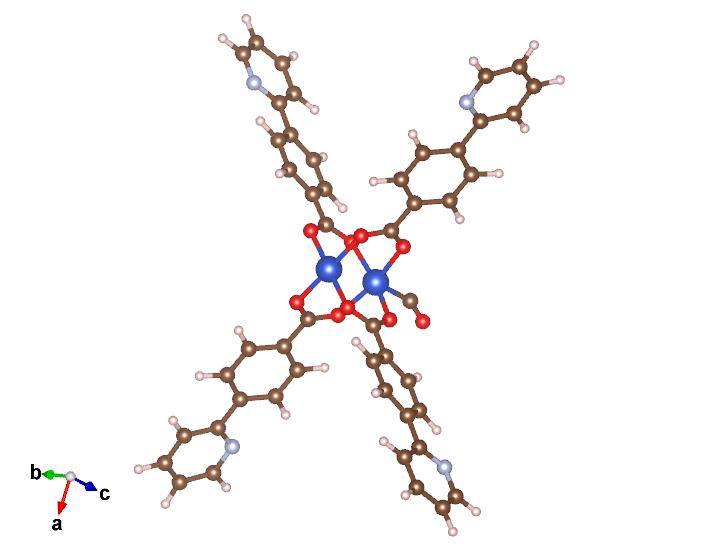
**

The lattice vector (Å) of the structural model.

| a | 30 | 0 | 0 |
| --- | --- | --- | --- |
| b | 0 | 30 | 0 |
| c | 0 | 0 | 30 |

**Supplementary Table 6.** Atomic coordinates for pristine Cu_2_(L)*CO

| O | 0.550337169 | 0.560964314 | 0.480624864 |
| --- | --- | --- | --- |
| O | 0.540281353 | 0.561145373 | 0.555950342 |
| O | 0.446955874 | 0.561628303 | 0.541803107 |
| O | 0.458852421 | 0.560261661 | 0.466735622 |
| O | 0.446609225 | 0.468226532 | 0.541519063 |
| O | 0.45922385 | 0.468978712 | 0.466555381 |
| O | 0.550729559 | 0.469112617 | 0.480433648 |
| O | 0.539929894 | 0.46778033 | 0.555695625 |
| O | 0.50775469 | 0.520065718 | 0.66136399 |
| C | 0.559414247 | 0.574039014 | 0.520221692 |
| C | 0.596416927 | 0.606917383 | 0.525396914 |
| C | 0.614942465 | 0.628210137 | 0.488176524 |
| C | 0.650083619 | 0.657944412 | 0.49354111 |
| C | 0.668032656 | 0.666740116 | 0.535969815 |
| C | 0.648966621 | 0.645370476 | 0.573063439 |
| C | 0.61352719 | 0.616082421 | 0.567916029 |
| C | 0.706303923 | 0.697321806 | 0.54262318 |
| C | 0.751367151 | 0.734299799 | 0.592990658 |
| C | 0.777417489 | 0.753261714 | 0.559380643 |
| C | 0.766715815 | 0.743069659 | 0.515309086 |
| C | 0.730937237 | 0.71480742 | 0.506776056 |
| C | 0.438879426 | 0.573910091 | 0.501922127 |
| C | 0.402175392 | 0.606935146 | 0.495621865 |
| C | 0.383443713 | 0.6279797 | 0.532875116 |
| C | 0.348277063 | 0.657652033 | 0.527720536 |
| C | 0.330416581 | 0.666808098 | 0.485348611 |
| C | 0.349880247 | 0.645943433 | 0.448108793 |
| C | 0.385392031 | 0.616573195 | 0.453068017 |
| C | 0.29191361 | 0.697271445 | 0.479184532 |
| C | 0.246257039 | 0.73463374 | 0.429597463 |
| C | 0.220516882 | 0.753337463 | 0.463585324 |
| C | 0.231672071 | 0.742785032 | 0.507464412 |
| C | 0.267547376 | 0.714471324 | 0.515401154 |
| C | 0.438740612 | 0.455819848 | 0.501634704 |
| C | 0.401715187 | 0.423166897 | 0.495263045 |
| C | 0.383202178 | 0.401779979 | 0.532426662 |
| C | 0.347925047 | 0.372280281 | 0.52717731 |
| C | 0.329680766 | 0.363744167 | 0.484838338 |
| C | 0.348815683 | 0.385075241 | 0.44770684 |
| C | 0.384439245 | 0.414266625 | 0.452758078 |
| C | 0.290985292 | 0.333540856 | 0.478661735 |
| C | 0.245316897 | 0.296132997 | 0.429164959 |
| C | 0.21934792 | 0.277823313 | 0.463199866 |
| C | 0.230332872 | 0.288681126 | 0.507040669 |
| C | 0.266316546 | 0.316857917 | 0.514889705 |
| C | 0.559625839 | 0.45575686 | 0.519978621 |
| C | 0.596999471 | 0.423358004 | 0.525074111 |
| C | 0.615435198 | 0.402040318 | 0.487849165 |
| C | 0.65067714 | 0.372441105 | 0.493190447 |
| C | 0.668835799 | 0.363880889 | 0.535590078 |
| C | 0.64996343 | 0.385473993 | 0.57266946 |
| C | 0.614414379 | 0.414628178 | 0.567557568 |
| C | 0.707143611 | 0.333342384 | 0.542261609 |
| C | 0.752149245 | 0.296136351 | 0.592548687 |
| C | 0.778256222 | 0.277283029 | 0.558933086 |
| C | 0.767670014 | 0.287743014 | 0.514902008 |
| C | 0.731947531 | 0.316095444 | 0.506406777 |
| C | 0.492224754 | 0.516545197 | 0.62667956 |
| H | 0.601434484 | 0.621159169 | 0.455183049 |
| H | 0.663453612 | 0.674490606 | 0.464061982 |
| H | 0.662822906 | 0.652301471 | 0.605902039 |
| H | 0.598853785 | 0.599415094 | 0.596673467 |
| H | 0.805260704 | 0.775143817 | 0.567659525 |
| H | 0.722587874 | 0.706160388 | 0.472506964 |
| H | 0.396948013 | 0.620675211 | 0.565787824 |
| H | 0.334819409 | 0.673879263 | 0.557321716 |
| H | 0.336224994 | 0.65328883 | 0.415249471 |
| H | 0.400190271 | 0.600343173 | 0.424094184 |
| H | 0.192623611 | 0.775320117 | 0.455757383 |
| H | 0.276251344 | 0.705618617 | 0.549526232 |
| H | 0.396967883 | 0.408698684 | 0.565316938 |
| H | 0.334693368 | 0.355690011 | 0.556666726 |
| H | 0.33481086 | 0.378266586 | 0.414875442 |
| H | 0.39903154 | 0.430897212 | 0.423904822 |
| H | 0.191398542 | 0.255904579 | 0.455433296 |
| H | 0.274911511 | 0.326020715 | 0.548969635 |
| H | 0.601692448 | 0.408891284 | 0.454915288 |
| H | 0.66394509 | 0.355786541 | 0.463730058 |
| H | 0.664021401 | 0.378777396 | 0.605477851 |
| H | 0.599829288 | 0.43143185 | 0.596277147 |
| H | 0.806043744 | 0.255330875 | 0.567176667 |
| H | 0.723739286 | 0.324978087 | 0.472150794 |
| H | 0.758705417 | 0.741139164 | 0.628086899 |
| H | 0.78616322 | 0.756821945 | 0.487788954 |
| H | 0.238678287 | 0.741867783 | 0.394627419 |
| H | 0.21256551 | 0.756343941 | 0.535307699 |
| H | 0.237807753 | 0.28872231 | 0.394221035 |
| H | 0.211046405 | 0.275459683 | 0.534922464 |
| H | 0.759330667 | 0.289085207 | 0.627628997 |
| H | 0.787179496 | 0.27413819 | 0.487354049 |
| N | 0.716867588 | 0.70722566 | 0.585273853 |
| N | 0.280901296 | 0.707522509 | 0.436736947 |
| N | 0.280083251 | 0.323097224 | 0.436233778 |
| N | 0.717610271 | 0.323206059 | 0.584889644 |
| Cu | 0.491984142 | 0.51460777 | 0.557903317 |
| Cu | 0.504879212 | 0.514780479 | 0.473498518 |

**Pristine Cu_2_(L)*OC(H)O**

**
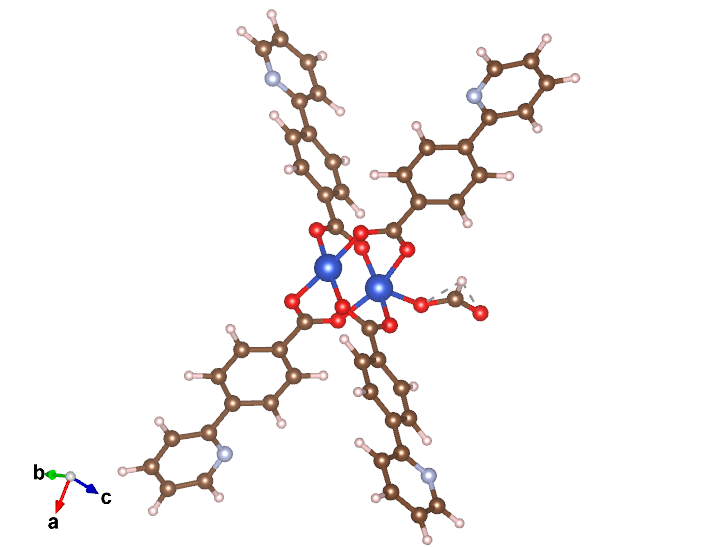
**

The lattice vector (Å) of the structural model.

| a | 30 | 0 | 0 |
| --- | --- | --- | --- |
| b | 0 | 30 | 0 |
| c | 0 | 0 | 30 |

**Supplementary Table 7.** Atomic coordinates for pristine Cu_2_(L)*OC(H)O

| O | 0.55145 | 0.560579 | 0.477917 |
| --- | --- | --- | --- |
| O | 0.542117 | 0.560327 | 0.553255 |
| O | 0.448336 | 0.561474 | 0.546843 |
| O | 0.459673 | 0.559303 | 0.471653 |
| O | 0.447469 | 0.468634 | 0.540481 |
| O | 0.459937 | 0.468694 | 0.465592 |
| O | 0.550532 | 0.469677 | 0.481948 |
| O | 0.540046 | 0.467373 | 0.557275 |
| O | 0.485476 | 0.510792 | 0.623715 |
| O | 0.465872 | 0.47487 | 0.684276 |
| C | 0.561006 | 0.573538 | 0.517542 |
| C | 0.597882 | 0.606505 | 0.522603 |
| C | 0.61694 | 0.627373 | 0.485387 |
| C | 0.651749 | 0.657508 | 0.490913 |
| C | 0.668752 | 0.667243 | 0.533533 |
| C | 0.649158 | 0.646212 | 0.570577 |
| C | 0.614137 | 0.616429 | 0.56532 |
| C | 0.706428 | 0.698539 | 0.540604 |
| C | 0.749599 | 0.736893 | 0.591653 |
| C | 0.776342 | 0.755695 | 0.55851 |
| C | 0.766924 | 0.744811 | 0.51432 |
| C | 0.73172 | 0.716 | 0.505199 |
| C | 0.439654 | 0.573281 | 0.507078 |
| C | 0.402816 | 0.605845 | 0.500221 |
| C | 0.383452 | 0.626993 | 0.53714 |
| C | 0.348398 | 0.65671 | 0.531223 |
| C | 0.331424 | 0.665837 | 0.488475 |
| C | 0.351339 | 0.64459 | 0.451697 |
| C | 0.386593 | 0.61511 | 0.457353 |
| C | 0.293517 | 0.696787 | 0.481119 |
| C | 0.250119 | 0.734602 | 0.429886 |
| C | 0.223587 | 0.753779 | 0.463002 |
| C | 0.233089 | 0.743208 | 0.507248 |
| C | 0.268256 | 0.714393 | 0.516491 |
| C | 0.439344 | 0.455852 | 0.500776 |
| C | 0.40215 | 0.423416 | 0.49466 |
| C | 0.383461 | 0.402233 | 0.531888 |
| C | 0.348124 | 0.372819 | 0.526633 |
| C | 0.329997 | 0.364214 | 0.484261 |
| C | 0.349285 | 0.385362 | 0.447099 |
| C | 0.38497 | 0.414428 | 0.452118 |
| C | 0.291378 | 0.333942 | 0.477996 |
| C | 0.246869 | 0.29528 | 0.428461 |
| C | 0.219985 | 0.278064 | 0.46236 |
| C | 0.229818 | 0.290211 | 0.50613 |
| C | 0.265721 | 0.318476 | 0.514071 |
| C | 0.559698 | 0.455719 | 0.521528 |
| C | 0.5971 | 0.423499 | 0.526011 |
| C | 0.615331 | 0.402278 | 0.488595 |
| C | 0.650734 | 0.372785 | 0.493656 |
| C | 0.669107 | 0.364168 | 0.53594 |
| C | 0.650343 | 0.385567 | 0.573213 |
| C | 0.614744 | 0.41472 | 0.568432 |
| C | 0.707448 | 0.333591 | 0.542332 |
| C | 0.751486 | 0.294869 | 0.592303 |
| C | 0.778265 | 0.277114 | 0.558597 |
| C | 0.768596 | 0.289018 | 0.514711 |
| C | 0.732963 | 0.317543 | 0.50643 |
| C | 0.466395 | 0.477384 | 0.642749 |
| H | 0.604103 | 0.619836 | 0.45224 |
| H | 0.665533 | 0.673711 | 0.461431 |
| H | 0.662151 | 0.653946 | 0.603609 |
| H | 0.599026 | 0.600188 | 0.594097 |
| H | 0.803607 | 0.778125 | 0.567233 |
| H | 0.724339 | 0.706917 | 0.470815 |
| H | 0.396295 | 0.619803 | 0.570357 |
| H | 0.334435 | 0.673051 | 0.56053 |
| H | 0.338316 | 0.651902 | 0.418591 |
| H | 0.401711 | 0.598691 | 0.428679 |
| H | 0.196297 | 0.776142 | 0.454197 |
| H | 0.27564 | 0.705457 | 0.550921 |
| H | 0.397026 | 0.409107 | 0.56489 |
| H | 0.334749 | 0.356278 | 0.556118 |
| H | 0.335408 | 0.378409 | 0.414245 |
| H | 0.399696 | 0.430923 | 0.423276 |
| H | 0.19213 | 0.256055 | 0.454504 |
| H | 0.273326 | 0.328816 | 0.548056 |
| H | 0.601364 | 0.409006 | 0.455729 |
| H | 0.663848 | 0.356107 | 0.464134 |
| H | 0.664494 | 0.378665 | 0.605945 |
| H | 0.600291 | 0.43135 | 0.597319 |
| H | 0.805868 | 0.254859 | 0.566668 |
| H | 0.725466 | 0.327635 | 0.472344 |
| H | 0.755683 | 0.744492 | 0.626828 |
| H | 0.786838 | 0.758562 | 0.487147 |
| H | 0.243756 | 0.741756 | 0.394666 |
| H | 0.2132 | 0.757137 | 0.534351 |
| H | 0.240189 | 0.286874 | 0.393574 |
| H | 0.209603 | 0.278052 | 0.533831 |
| H | 0.757966 | 0.286674 | 0.627278 |
| H | 0.788702 | 0.276359 | 0.487147 |
| H | 0.450509 | 0.451397 | 0.621704 |
| N | 0.715688 | 0.709231 | 0.583368 |
| N | 0.284082 | 0.707024 | 0.438277 |
| N | 0.28157 | 0.322309 | 0.435605 |
| N | 0.71712 | 0.322206 | 0.584828 |
| Cu | 0.49387 | 0.514606 | 0.557663 |
| Cu | 0.505305 | 0.514546 | 0.474335 |

**Pristine Cu_2_(L)*OC(H)OH**

**
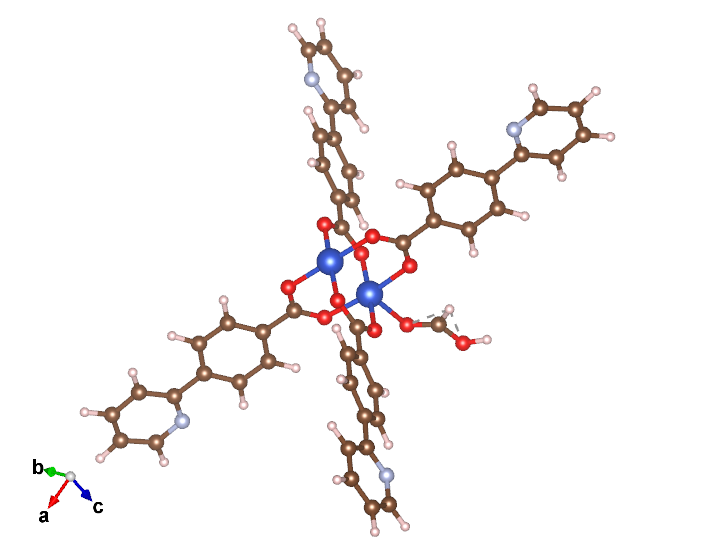
**

The lattice vector (Å) of the structural model.

| a | 30 | 0 | 0 |
| --- | --- | --- | --- |
| b | 0 | 30 | 0 |
| c | 0 | 0 | 30 |

**Supplementary Table 8.** Atomic coordinates for pristine Cu_2_(L)*OC(H)OH

| O | 0.551819 | 0.56073 | 0.478462 |
| --- | --- | --- | --- |
| O | 0.541844 | 0.560773 | 0.553808 |
| O | 0.448173 | 0.5606 | 0.543756 |
| O | 0.459381 | 0.561027 | 0.468558 |
| O | 0.448178 | 0.467535 | 0.542687 |
| O | 0.459092 | 0.469032 | 0.467507 |
| O | 0.551506 | 0.468543 | 0.480359 |
| O | 0.540617 | 0.468056 | 0.555634 |
| O | 0.484559 | 0.512901 | 0.630215 |
| O | 0.46019 | 0.473949 | 0.688768 |
| C | 0.561061 | 0.573672 | 0.518066 |
| C | 0.598113 | 0.606518 | 0.523293 |
| C | 0.617309 | 0.627096 | 0.486 |
| C | 0.652216 | 0.657147 | 0.491304 |
| C | 0.669211 | 0.667168 | 0.533843 |
| C | 0.649551 | 0.646416 | 0.571016 |
| C | 0.614439 | 0.616688 | 0.565906 |
| C | 0.706848 | 0.698535 | 0.540639 |
| C | 0.749969 | 0.737647 | 0.591187 |
| C | 0.776508 | 0.756298 | 0.557801 |
| C | 0.767063 | 0.744866 | 0.513752 |
| C | 0.732005 | 0.715766 | 0.505013 |
| C | 0.439572 | 0.573743 | 0.504184 |
| C | 0.402512 | 0.606475 | 0.498778 |
| C | 0.383287 | 0.627032 | 0.536094 |
| C | 0.348088 | 0.656755 | 0.530814 |
| C | 0.330799 | 0.666369 | 0.488279 |
| C | 0.350509 | 0.645658 | 0.45112 |
| C | 0.38594 | 0.61632 | 0.456213 |
| C | 0.292862 | 0.697318 | 0.481345 |
| C | 0.249442 | 0.735567 | 0.430388 |
| C | 0.222945 | 0.754549 | 0.463614 |
| C | 0.232485 | 0.743644 | 0.507787 |
| C | 0.267623 | 0.714735 | 0.516834 |
| C | 0.439311 | 0.455499 | 0.502754 |
| C | 0.402043 | 0.423136 | 0.496377 |
| C | 0.383035 | 0.401601 | 0.533172 |
| C | 0.347686 | 0.372246 | 0.527507 |
| C | 0.329883 | 0.363961 | 0.484937 |
| C | 0.349458 | 0.385388 | 0.448126 |
| C | 0.385082 | 0.41446 | 0.453687 |
| C | 0.291313 | 0.333803 | 0.478263 |
| C | 0.247294 | 0.294905 | 0.428506 |
| C | 0.220078 | 0.27786 | 0.462223 |
| C | 0.229457 | 0.290278 | 0.506011 |
| C | 0.26526 | 0.318588 | 0.51414 |
| C | 0.560365 | 0.455547 | 0.520001 |
| C | 0.597639 | 0.423071 | 0.525393 |
| C | 0.615964 | 0.401551 | 0.488218 |
| C | 0.65113 | 0.371858 | 0.493499 |
| C | 0.669303 | 0.363357 | 0.53586 |
| C | 0.65056 | 0.385061 | 0.572953 |
| C | 0.615126 | 0.414384 | 0.567857 |
| C | 0.707637 | 0.332852 | 0.542436 |
| C | 0.75167 | 0.294137 | 0.592414 |
| C | 0.778653 | 0.276651 | 0.558744 |
| C | 0.769148 | 0.288699 | 0.514866 |
| C | 0.733432 | 0.317121 | 0.506584 |
| C | 0.465847 | 0.479916 | 0.644492 |
| H | 0.60441 | 0.619298 | 0.452931 |
| H | 0.66603 | 0.673114 | 0.461695 |
| H | 0.662607 | 0.65434 | 0.603981 |
| H | 0.599318 | 0.600607 | 0.594772 |
| H | 0.803687 | 0.778955 | 0.566208 |
| H | 0.724577 | 0.706288 | 0.470738 |
| H | 0.396454 | 0.619464 | 0.569105 |
| H | 0.334299 | 0.672768 | 0.560397 |
| H | 0.337209 | 0.653311 | 0.418203 |
| H | 0.401083 | 0.600271 | 0.427356 |
| H | 0.19571 | 0.777041 | 0.454977 |
| H | 0.275084 | 0.705636 | 0.551208 |
| H | 0.396463 | 0.408249 | 0.566267 |
| H | 0.334106 | 0.355445 | 0.556732 |
| H | 0.335811 | 0.378734 | 0.415111 |
| H | 0.400026 | 0.431241 | 0.425124 |
| H | 0.192291 | 0.255812 | 0.454215 |
| H | 0.272555 | 0.329152 | 0.548115 |
| H | 0.602134 | 0.408389 | 0.455311 |
| H | 0.664306 | 0.354999 | 0.464098 |
| H | 0.664733 | 0.378417 | 0.605742 |
| H | 0.60079 | 0.431458 | 0.596566 |
| H | 0.806323 | 0.254485 | 0.566851 |
| H | 0.726071 | 0.327413 | 0.47253 |
| H | 0.756144 | 0.745604 | 0.626276 |
| H | 0.786867 | 0.758389 | 0.486375 |
| H | 0.243218 | 0.743067 | 0.395219 |
| H | 0.21264 | 0.757474 | 0.53499 |
| H | 0.240921 | 0.286341 | 0.393601 |
| H | 0.208923 | 0.27833 | 0.53358 |
| H | 0.758128 | 0.2859 | 0.627383 |
| H | 0.789446 | 0.276281 | 0.487336 |
| H | 0.452344 | 0.453186 | 0.62283 |
| H | 0.44453 | 0.445866 | 0.694042 |
| N | 0.716213 | 0.709693 | 0.583286 |
| N | 0.283355 | 0.70791 | 0.438587 |
| N | 0.281931 | 0.321972 | 0.435834 |
| N | 0.717172 | 0.321315 | 0.584928 |
| Cu | 0.494432 | 0.514971 | 0.554376 |
| Cu | 0.505558 | 0.514791 | 0.472452 |

**Defect Cu_2_(L)*COOH**

**
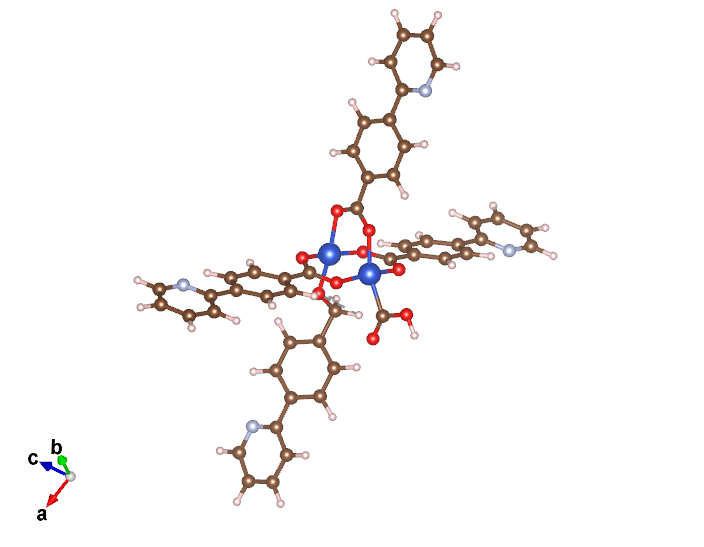
**

The lattice vector (Å) of the structural model.

| a | 30 | 0 | 0 |
| --- | --- | --- | --- |
| b | 0 | 30 | 0 |
| c | 0 | 0 | 30 |

**Supplementary Supplementary Table 9.** Atomic coordinates for defect Cu_2_(L)*COOH

| O | 0.551835 | 0.566771 | 0.465051 |
| --- | --- | --- | --- |
| O | 0.539162 | 0.566776 | 0.540407 |
| O | 0.446757 | 0.563183 | 0.545626 |
| O | 0.457469 | 0.573628 | 0.471729 |
| O | 0.450794 | 0.473999 | 0.526182 |
| O | 0.455241 | 0.482465 | 0.450423 |
| O | 0.542724 | 0.478846 | 0.53962 |
| O | 0.564942 | 0.465255 | 0.422606 |
| O | 0.524688 | 0.506717 | 0.373195 |
| C | 0.560265 | 0.578477 | 0.505613 |
| C | 0.598812 | 0.609187 | 0.512416 |
| C | 0.620743 | 0.629294 | 0.476405 |
| C | 0.656559 | 0.657782 | 0.483971 |
| C | 0.671709 | 0.666638 | 0.527463 |
| C | 0.649508 | 0.646142 | 0.563284 |
| C | 0.613582 | 0.617928 | 0.555947 |
| C | 0.709922 | 0.69675 | 0.536532 |
| C | 0.751293 | 0.734475 | 0.589486 |
| C | 0.779644 | 0.753062 | 0.557557 |
| C | 0.772248 | 0.742102 | 0.513002 |
| C | 0.73721 | 0.713619 | 0.502331 |
| C | 0.436753 | 0.580977 | 0.508333 |
| C | 0.398093 | 0.611995 | 0.505416 |
| C | 0.376692 | 0.628183 | 0.543639 |
| C | 0.340486 | 0.656943 | 0.539616 |
| C | 0.324434 | 0.669906 | 0.497488 |
| C | 0.345927 | 0.653047 | 0.459389 |
| C | 0.382299 | 0.624784 | 0.463267 |
| C | 0.285951 | 0.700459 | 0.491654 |
| C | 0.242153 | 0.740161 | 0.442186 |
| C | 0.215124 | 0.75717 | 0.476042 |
| C | 0.224639 | 0.744513 | 0.519738 |
| C | 0.260297 | 0.715922 | 0.527701 |
| C | 0.438982 | 0.465306 | 0.48628 |
| C | 0.402582 | 0.431962 | 0.481147 |
| C | 0.386988 | 0.409357 | 0.519016 |
| C | 0.352623 | 0.378581 | 0.515355 |
| C | 0.332535 | 0.369797 | 0.473932 |
| C | 0.348607 | 0.392354 | 0.436097 |
| C | 0.383209 | 0.42301 | 0.439546 |
| C | 0.295311 | 0.337584 | 0.469495 |
| C | 0.253231 | 0.293316 | 0.422636 |
| C | 0.227052 | 0.277152 | 0.457585 |
| C | 0.235824 | 0.292947 | 0.500411 |
| C | 0.270118 | 0.323586 | 0.50648 |
| C | 0.537024 | 0.433281 | 0.529308 |
| C | 0.581901 | 0.410707 | 0.533855 |
| C | 0.602896 | 0.391423 | 0.496919 |
| C | 0.642883 | 0.368321 | 0.501776 |
| C | 0.663353 | 0.364269 | 0.543717 |
| C | 0.642757 | 0.384836 | 0.580516 |
| C | 0.602834 | 0.407781 | 0.57556 |
| C | 0.704662 | 0.338002 | 0.550146 |
| C | 0.751205 | 0.301811 | 0.599761 |
| C | 0.779412 | 0.286619 | 0.566001 |
| C | 0.769415 | 0.29888 | 0.522288 |
| C | 0.731897 | 0.324914 | 0.514225 |
| C | 0.53734 | 0.493248 | 0.414504 |
| H | 0.609266 | 0.622519 | 0.442597 |
| H | 0.672496 | 0.673484 | 0.455316 |
| H | 0.661152 | 0.653066 | 0.596984 |
| H | 0.596493 | 0.602033 | 0.583779 |
| H | 0.806694 | 0.775243 | 0.567478 |
| H | 0.731411 | 0.704414 | 0.467667 |
| H | 0.388862 | 0.618171 | 0.576391 |
| H | 0.324823 | 0.669466 | 0.569941 |
| H | 0.333372 | 0.662963 | 0.426784 |
| H | 0.399004 | 0.611957 | 0.433674 |
| H | 0.18757 | 0.779571 | 0.468227 |
| H | 0.267881 | 0.705643 | 0.561701 |
| H | 0.402378 | 0.416173 | 0.551197 |
| H | 0.341775 | 0.360799 | 0.545161 |
| H | 0.333237 | 0.385149 | 0.403967 |
| H | 0.395409 | 0.440574 | 0.410166 |
| H | 0.200472 | 0.253153 | 0.451257 |
| H | 0.276832 | 0.336917 | 0.539613 |
| H | 0.587457 | 0.394295 | 0.464128 |
| H | 0.657547 | 0.352429 | 0.472533 |
| H | 0.65857 | 0.381612 | 0.613069 |
| H | 0.587126 | 0.423547 | 0.604363 |
| H | 0.808359 | 0.266084 | 0.573923 |
| H | 0.723983 | 0.335257 | 0.4803 |
| H | 0.75597 | 0.741926 | 0.624905 |
| H | 0.793604 | 0.755461 | 0.486738 |
| H | 0.235887 | 0.748994 | 0.407324 |
| H | 0.204476 | 0.756785 | 0.547434 |
| H | 0.247282 | 0.282105 | 0.388412 |
| H | 0.215977 | 0.281759 | 0.52879 |
| H | 0.758051 | 0.293477 | 0.634642 |
| H | 0.790694 | 0.288294 | 0.494721 |
| H | 0.525165 | 0.42825 | 0.494744 |
| H | 0.512856 | 0.416968 | 0.552011 |
| H | 0.541797 | 0.489198 | 0.351197 |
| N | 0.717411 | 0.70726 | 0.579682 |
| N | 0.276501 | 0.712744 | 0.449355 |
| N | 0.286437 | 0.322575 | 0.427964 |
| N | 0.714906 | 0.326612 | 0.592542 |
| Cu | 0.495303 | 0.518564 | 0.539731 |
| Cu | 0.500816 | 0.528908 | 0.4543 |

**Defect Cu_2_(L)*CO**

**
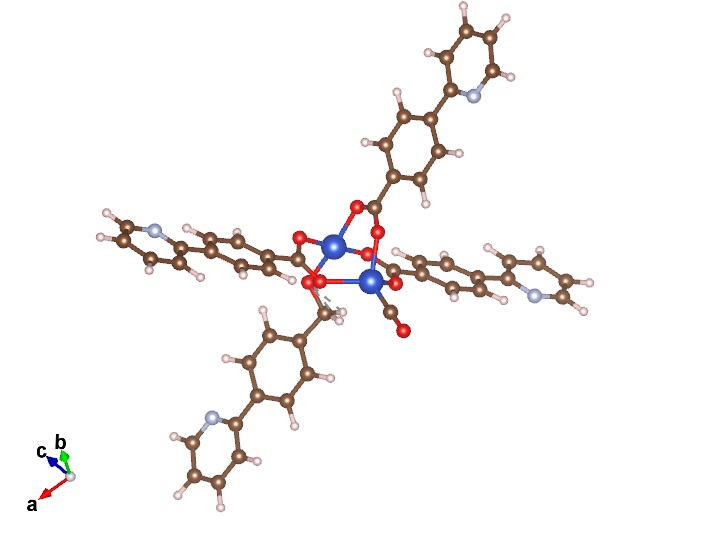
**

The lattice vector (Å) of the structural model.

| a | 30 | 0 | 0 |
| --- | --- | --- | --- |
| b | 0 | 30 | 0 |
| c | 0 | 0 | 30 |

**Supplementary Supplementary Table 10.** Atomic coordinates for defect Cu_2_(L)*CO

| O | 0.558213 | 0.550039 | 0.477428 |
| --- | --- | --- | --- |
| O | 0.535134 | 0.564445 | 0.548156 |
| O | 0.446283 | 0.55886 | 0.53826 |
| O | 0.461633 | 0.575879 | 0.465931 |
| O | 0.452945 | 0.470684 | 0.537131 |
| O | 0.4609 | 0.475552 | 0.462014 |
| O | 0.541431 | 0.478646 | 0.550871 |
| O | 0.522736 | 0.518885 | 0.347929 |
| C | 0.562149 | 0.569055 | 0.514791 |
| C | 0.599714 | 0.600521 | 0.522352 |
| C | 0.61992 | 0.620986 | 0.485531 |
| C | 0.654441 | 0.651188 | 0.491739 |
| C | 0.670233 | 0.661356 | 0.534749 |
| C | 0.649822 | 0.640417 | 0.5714 |
| C | 0.61482 | 0.610652 | 0.565395 |
| C | 0.707368 | 0.693189 | 0.542158 |
| C | 0.749775 | 0.732522 | 0.593069 |
| C | 0.775647 | 0.752392 | 0.559847 |
| C | 0.766337 | 0.741341 | 0.515674 |
| C | 0.732053 | 0.711429 | 0.506681 |
| C | 0.439061 | 0.579689 | 0.501238 |
| C | 0.399933 | 0.61035 | 0.499636 |
| C | 0.37938 | 0.627487 | 0.537878 |
| C | 0.343491 | 0.656699 | 0.533856 |
| C | 0.326771 | 0.668925 | 0.491779 |
| C | 0.347348 | 0.650919 | 0.453692 |
| C | 0.383459 | 0.622379 | 0.457545 |
| C | 0.288679 | 0.699953 | 0.485901 |
| C | 0.244803 | 0.739075 | 0.435994 |
| C | 0.218652 | 0.757546 | 0.469777 |
| C | 0.228635 | 0.74591 | 0.513658 |
| C | 0.263859 | 0.716848 | 0.521895 |
| C | 0.442866 | 0.45962 | 0.496558 |
| C | 0.406686 | 0.425853 | 0.492106 |
| C | 0.388009 | 0.405326 | 0.529717 |
| C | 0.352787 | 0.375574 | 0.524997 |
| C | 0.334937 | 0.365739 | 0.48275 |
| C | 0.354471 | 0.385954 | 0.445198 |
| C | 0.389873 | 0.415452 | 0.449734 |
| C | 0.296151 | 0.33557 | 0.476832 |
| C | 0.250771 | 0.297724 | 0.42741 |
| C | 0.223696 | 0.280992 | 0.461377 |
| C | 0.234133 | 0.292662 | 0.50514 |
| C | 0.270609 | 0.320211 | 0.513029 |
| C | 0.545361 | 0.442048 | 0.521794 |
| C | 0.589274 | 0.418137 | 0.528997 |
| C | 0.609253 | 0.396866 | 0.492502 |
| C | 0.647017 | 0.370571 | 0.498026 |
| C | 0.666808 | 0.365376 | 0.54021 |
| C | 0.647424 | 0.387998 | 0.576407 |
| C | 0.609321 | 0.413999 | 0.570966 |
| C | 0.706516 | 0.336757 | 0.54699 |
| C | 0.751844 | 0.299669 | 0.597099 |
| C | 0.778712 | 0.281957 | 0.563467 |
| C | 0.768628 | 0.29327 | 0.519512 |
| C | 0.732441 | 0.321023 | 0.511124 |
| C | 0.51537 | 0.521839 | 0.385595 |
| H | 0.607836 | 0.613076 | 0.452177 |
| H | 0.668825 | 0.667263 | 0.462495 |
| H | 0.662092 | 0.648264 | 0.604686 |
| H | 0.599003 | 0.594662 | 0.593942 |
| H | 0.802207 | 0.775679 | 0.568477 |
| H | 0.724724 | 0.702356 | 0.472295 |
| H | 0.392029 | 0.618192 | 0.570666 |
| H | 0.328698 | 0.670245 | 0.564186 |
| H | 0.334385 | 0.660332 | 0.421094 |
| H | 0.399661 | 0.608861 | 0.427968 |
| H | 0.191331 | 0.780142 | 0.461725 |
| H | 0.271768 | 0.707318 | 0.556046 |
| H | 0.401245 | 0.413149 | 0.562635 |
| H | 0.339241 | 0.359871 | 0.554843 |
| H | 0.340848 | 0.37809 | 0.412445 |
| H | 0.404645 | 0.431442 | 0.420617 |
| H | 0.195339 | 0.259595 | 0.453602 |
| H | 0.278788 | 0.329998 | 0.547043 |
| H | 0.594395 | 0.400326 | 0.459423 |
| H | 0.660649 | 0.353412 | 0.468993 |
| H | 0.662783 | 0.384304 | 0.60915 |
| H | 0.594623 | 0.431234 | 0.599425 |
| H | 0.806696 | 0.260222 | 0.571659 |
| H | 0.724595 | 0.330658 | 0.476987 |
| H | 0.755936 | 0.740104 | 0.628227 |
| H | 0.785585 | 0.755816 | 0.488398 |
| H | 0.238185 | 0.747057 | 0.400991 |
| H | 0.209035 | 0.759259 | 0.541259 |
| H | 0.243866 | 0.28944 | 0.392539 |
| H | 0.213972 | 0.280586 | 0.532915 |
| H | 0.758777 | 0.292104 | 0.632142 |
| H | 0.788826 | 0.280671 | 0.491984 |
| H | 0.544556 | 0.452712 | 0.486488 |
| H | 0.517965 | 0.417701 | 0.526644 |
| N | 0.716641 | 0.703848 | 0.584926 |
| N | 0.278731 | 0.711211 | 0.443428 |
| N | 0.28592 | 0.324163 | 0.434476 |
| N | 0.716779 | 0.326133 | 0.589571 |
| Cu | 0.494647 | 0.517192 | 0.54163 |
| Cu | 0.502618 | 0.52849 | 0.443828 |

**Defect Cu_2_(L)*OOCH**

**
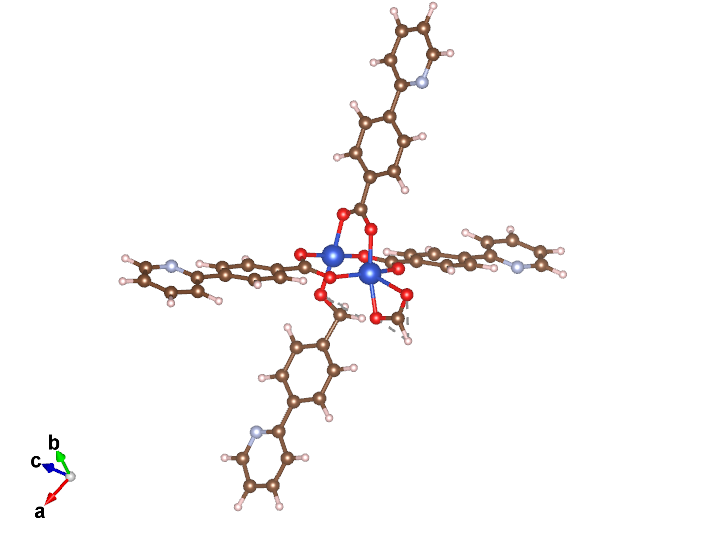
**

The lattice vector (Å) of the structural model.

| a | 30 | 0 | 0 |
| --- | --- | --- | --- |
| b | 0 | 30 | 0 |
| c | 0 | 0 | 30 |

**Supplementary Supplementary Table 11.** Atomic coordinates for defect Cu_2_(L)*OOCH

| O | 0.554194 | 0.56421 | 0.46668 |
| --- | --- | --- | --- |
| O | 0.538755 | 0.564512 | 0.541226 |
| O | 0.448932 | 0.562614 | 0.533368 |
| O | 0.460239 | 0.575264 | 0.459508 |
| O | 0.45193 | 0.47358 | 0.529769 |
| O | 0.452841 | 0.481857 | 0.454233 |
| O | 0.541605 | 0.478829 | 0.538518 |
| O | 0.541186 | 0.480146 | 0.422424 |
| O | 0.506839 | 0.520083 | 0.370016 |
| C | 0.561542 | 0.576064 | 0.506616 |
| C | 0.59966 | 0.60672 | 0.515447 |
| C | 0.620321 | 0.628904 | 0.479878 |
| C | 0.655869 | 0.657573 | 0.487807 |
| C | 0.671808 | 0.664733 | 0.53132 |
| C | 0.650739 | 0.642357 | 0.566725 |
| C | 0.615164 | 0.613759 | 0.558997 |
| C | 0.709753 | 0.695019 | 0.540788 |
| C | 0.749462 | 0.734191 | 0.593916 |
| C | 0.778997 | 0.751568 | 0.562431 |
| C | 0.773175 | 0.739148 | 0.518008 |
| C | 0.738379 | 0.710508 | 0.507024 |
| C | 0.43976 | 0.58176 | 0.496077 |
| C | 0.401451 | 0.613266 | 0.495616 |
| C | 0.381948 | 0.628473 | 0.535271 |
| C | 0.345509 | 0.65709 | 0.533774 |
| C | 0.327295 | 0.670947 | 0.492814 |
| C | 0.347269 | 0.65554 | 0.453292 |
| C | 0.38387 | 0.62724 | 0.45458 |
| C | 0.288164 | 0.701129 | 0.48992 |
| C | 0.242218 | 0.742738 | 0.444102 |
| C | 0.216123 | 0.757534 | 0.479713 |
| C | 0.227188 | 0.742644 | 0.52232 |
| C | 0.263396 | 0.714082 | 0.527543 |
| C | 0.438278 | 0.464486 | 0.489809 |
| C | 0.402436 | 0.430379 | 0.48628 |
| C | 0.386135 | 0.408322 | 0.524113 |
| C | 0.352219 | 0.377013 | 0.519959 |
| C | 0.333524 | 0.367118 | 0.478114 |
| C | 0.35062 | 0.388975 | 0.440313 |
| C | 0.384593 | 0.42016 | 0.444263 |
| C | 0.296276 | 0.335024 | 0.473018 |
| C | 0.25407 | 0.29206 | 0.42507 |
| C | 0.226795 | 0.276414 | 0.459393 |
| C | 0.235261 | 0.291548 | 0.502536 |
| C | 0.270245 | 0.321185 | 0.509488 |
| C | 0.535924 | 0.434145 | 0.524481 |
| C | 0.581341 | 0.413117 | 0.529754 |
| C | 0.605752 | 0.399336 | 0.492405 |
| C | 0.645559 | 0.376067 | 0.497708 |
| C | 0.662735 | 0.367046 | 0.540443 |
| C | 0.639089 | 0.383121 | 0.577691 |
| C | 0.599147 | 0.405863 | 0.572402 |
| C | 0.703938 | 0.340659 | 0.547423 |
| C | 0.750316 | 0.305771 | 0.598219 |
| C | 0.778905 | 0.290032 | 0.565022 |
| C | 0.768966 | 0.300986 | 0.52095 |
| C | 0.731267 | 0.326519 | 0.511992 |
| C | 0.532208 | 0.488921 | 0.381127 |
| H | 0.607988 | 0.623474 | 0.446127 |
| H | 0.67088 | 0.675045 | 0.459674 |
| H | 0.663043 | 0.648098 | 0.600427 |
| H | 0.599052 | 0.596434 | 0.586552 |
| H | 0.805789 | 0.773946 | 0.572594 |
| H | 0.733882 | 0.699876 | 0.472564 |
| H | 0.395716 | 0.617746 | 0.567135 |
| H | 0.331414 | 0.668872 | 0.565148 |
| H | 0.333277 | 0.66642 | 0.421584 |
| H | 0.399262 | 0.615299 | 0.423894 |
| H | 0.187969 | 0.77984 | 0.474025 |
| H | 0.272115 | 0.70187 | 0.560624 |
| H | 0.400342 | 0.415966 | 0.55665 |
| H | 0.340544 | 0.35986 | 0.54981 |
| H | 0.336424 | 0.380893 | 0.407849 |
| H | 0.397658 | 0.437325 | 0.415013 |
| H | 0.199673 | 0.25324 | 0.452328 |
| H | 0.276803 | 0.333981 | 0.542848 |
| H | 0.592693 | 0.406252 | 0.459134 |
| H | 0.662806 | 0.364177 | 0.467974 |
| H | 0.652673 | 0.376538 | 0.61074 |
| H | 0.580904 | 0.417862 | 0.601524 |
| H | 0.808028 | 0.270014 | 0.573607 |
| H | 0.723363 | 0.335444 | 0.47768 |
| H | 0.752947 | 0.742772 | 0.629214 |
| H | 0.795643 | 0.751494 | 0.492182 |
| H | 0.234621 | 0.753291 | 0.409993 |
| H | 0.207759 | 0.75301 | 0.551291 |
| H | 0.248529 | 0.281212 | 0.390678 |
| H | 0.214687 | 0.280594 | 0.530481 |
| H | 0.756888 | 0.298254 | 0.633331 |
| H | 0.790362 | 0.289752 | 0.493736 |
| H | 0.525954 | 0.432079 | 0.489138 |
| H | 0.511295 | 0.416249 | 0.545229 |
| H | 0.548324 | 0.466999 | 0.356062 |
| N | 0.715768 | 0.70683 | 0.583839 |
| N | 0.277168 | 0.715533 | 0.448672 |
| N | 0.287854 | 0.320462 | 0.431258 |
| N | 0.713997 | 0.330216 | 0.59011 |
| Cu | 0.495556 | 0.518729 | 0.534403 |
| Cu | 0.502395 | 0.5272 | 0.44757 |

**Defect Cu_2_(L)*OC(H)OH**

**
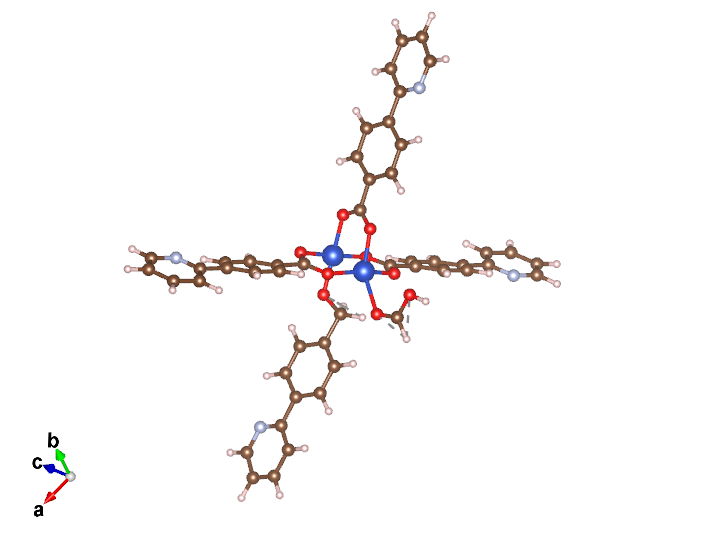
**

The lattice vector (Å) of the structural model.

| a | 30 | 0 | 0 |
| --- | --- | --- | --- |
| b | 0 | 30 | 0 |
| c | 0 | 0 | 30 |

**Supplementary Supplementary Table 12.** Atomic coordinates for defect Cu_2_(L)*OC(H)OH

| O | 0.554414 | 0.563728 | 0.469773 |
| --- | --- | --- | --- |
| O | 0.539513 | 0.5666 | 0.544306 |
| O | 0.44806 | 0.565431 | 0.536098 |
| O | 0.457478 | 0.573467 | 0.461148 |
| O | 0.448315 | 0.474592 | 0.530854 |
| O | 0.458358 | 0.477662 | 0.455586 |
| O | 0.541845 | 0.47919 | 0.535306 |
| O | 0.541932 | 0.485929 | 0.410619 |
| O | 0.506597 | 0.517855 | 0.353305 |
| C | 0.562016 | 0.576818 | 0.509683 |
| C | 0.600699 | 0.607338 | 0.516869 |
| C | 0.621092 | 0.629206 | 0.480977 |
| C | 0.656621 | 0.658118 | 0.488354 |
| C | 0.672978 | 0.665592 | 0.531653 |
| C | 0.652433 | 0.643243 | 0.567372 |
| C | 0.616736 | 0.614654 | 0.560165 |
| C | 0.710776 | 0.696126 | 0.540675 |
| C | 0.750171 | 0.736303 | 0.593318 |
| C | 0.780083 | 0.752904 | 0.561766 |
| C | 0.77454 | 0.739602 | 0.517549 |
| C | 0.739722 | 0.710852 | 0.506845 |
| C | 0.438311 | 0.582182 | 0.498086 |
| C | 0.400256 | 0.614287 | 0.496611 |
| C | 0.380357 | 0.629706 | 0.53595 |
| C | 0.34415 | 0.658599 | 0.534011 |
| C | 0.326519 | 0.672548 | 0.492845 |
| C | 0.346914 | 0.65703 | 0.453584 |
| C | 0.38333 | 0.628491 | 0.45542 |
| C | 0.287395 | 0.702661 | 0.4898 |
| C | 0.241013 | 0.74407 | 0.444271 |
| C | 0.214692 | 0.758285 | 0.479929 |
| C | 0.226031 | 0.743417 | 0.522487 |
| C | 0.262608 | 0.715359 | 0.527536 |
| C | 0.439295 | 0.46295 | 0.490807 |
| C | 0.403217 | 0.429147 | 0.4856 |
| C | 0.386187 | 0.40729 | 0.523256 |
| C | 0.351963 | 0.376409 | 0.518879 |
| C | 0.333395 | 0.366807 | 0.476929 |
| C | 0.350887 | 0.388649 | 0.439308 |
| C | 0.385418 | 0.419275 | 0.443533 |
| C | 0.296078 | 0.334857 | 0.47177 |
| C | 0.254181 | 0.291238 | 0.424125 |
| C | 0.226863 | 0.275768 | 0.458517 |
| C | 0.235078 | 0.291431 | 0.501498 |
| C | 0.269846 | 0.321376 | 0.508221 |
| C | 0.535438 | 0.433285 | 0.526826 |
| C | 0.580252 | 0.410861 | 0.531539 |
| C | 0.60432 | 0.396664 | 0.494166 |
| C | 0.644696 | 0.374202 | 0.498968 |
| C | 0.662607 | 0.365737 | 0.541438 |
| C | 0.639007 | 0.381483 | 0.578832 |
| C | 0.598659 | 0.403653 | 0.573869 |
| C | 0.704376 | 0.340135 | 0.548028 |
| C | 0.751644 | 0.305555 | 0.59824 |
| C | 0.78033 | 0.290546 | 0.564819 |
| C | 0.769953 | 0.301777 | 0.520917 |
| C | 0.731863 | 0.326847 | 0.512383 |
| C | 0.534418 | 0.487423 | 0.370746 |
| H | 0.608412 | 0.623516 | 0.447395 |
| H | 0.671237 | 0.675528 | 0.45997 |
| H | 0.665124 | 0.649073 | 0.600907 |
| H | 0.600778 | 0.59743 | 0.587888 |
| H | 0.806973 | 0.775284 | 0.571695 |
| H | 0.735439 | 0.699508 | 0.472596 |
| H | 0.393706 | 0.618722 | 0.567911 |
| H | 0.32973 | 0.670367 | 0.565239 |
| H | 0.333336 | 0.667934 | 0.421695 |
| H | 0.39909 | 0.616402 | 0.424996 |
| H | 0.186257 | 0.780258 | 0.474367 |
| H | 0.271518 | 0.7032 | 0.560575 |
| H | 0.400298 | 0.414842 | 0.555864 |
| H | 0.340017 | 0.359223 | 0.548607 |
| H | 0.336603 | 0.380957 | 0.406797 |
| H | 0.398771 | 0.436438 | 0.414409 |
| H | 0.199908 | 0.252356 | 0.451632 |
| H | 0.276145 | 0.334653 | 0.541442 |
| H | 0.590786 | 0.402892 | 0.460921 |
| H | 0.661893 | 0.362656 | 0.469052 |
| H | 0.652918 | 0.375182 | 0.611789 |
| H | 0.580641 | 0.415489 | 0.603214 |
| H | 0.809707 | 0.270763 | 0.57305 |
| H | 0.723707 | 0.3361 | 0.478207 |
| H | 0.753401 | 0.74559 | 0.628472 |
| H | 0.797273 | 0.751297 | 0.491652 |
| H | 0.233383 | 0.754897 | 0.410257 |
| H | 0.206514 | 0.753412 | 0.551535 |
| H | 0.248712 | 0.280077 | 0.389808 |
| H | 0.214441 | 0.280703 | 0.529485 |
| H | 0.758516 | 0.297842 | 0.633263 |
| H | 0.791364 | 0.291145 | 0.493465 |
| H | 0.522546 | 0.427072 | 0.492819 |
| H | 0.51173 | 0.41813 | 0.550734 |
| H | 0.549906 | 0.464067 | 0.346736 |
| H | 0.504514 | 0.514386 | 0.320951 |
| N | 0.716469 | 0.708867 | 0.583508 |
| N | 0.27627 | 0.717169 | 0.44862 |
| N | 0.287863 | 0.319819 | 0.430117 |
| N | 0.714866 | 0.329459 | 0.59055 |
| Cu | 0.494713 | 0.519358 | 0.538235 |
| Cu | 0.503526 | 0.525689 | 0.456304 |

**Supplementary References**

1 Zhu, Q. G. *et al.* Efficient Reduction of CO_2_ into formic acid on a lead or tin electrode using an ionic liquid catholyte mixture. *Angew. Chem. Int. Ed.* **55**, 9012-9016 (2016).

2 Sun, X. F. *et al.* MoP nanoparticles supported on indium-doped porous carbon: outstanding catalysts for highly efficient CO_2_ electroreduction. *Angew. Chem. Int. Ed.* **57**, 2427-2431 (2018).

3 Wu, H. R. *et al.* Design of naturally derived lead phytate as an electrocatalyst for highly efficient CO_2_ reduction to formic acid. *Green Chem.* **20**, 4602-4606 (2018).

4 Lu, L. *et al.* Selective electroreduction of carbon dioxide to formic acid on electrodeposited SnO_2_@N-doped porous carbon catalysts. *Sci. China Chem.* **61**, 228-235 (2018).

5 Wu, J. X. *et al.* Cathodized copper porphyrin metal-organic framework nanosheets for selective formate and acetate production from CO_2_ electroreduction. *Chem. Sci.* **10**, 2199-2205 (2019).

6 Zhang, X. J. *et al.* Electrochemical reduction of carbon dioxide to formic acid in ionic liquid [Emim][N(CN)_2_]/water system. *Electrochim. Acta* **247**, 281-287 (2017).

7 Atifi, A., Boyce, D. W., DiMeglio, J. L. & Rosenthal, J. Directing the outcome of CO_2_ reduction at bismuth cathodes using varied ionic liquid promoters. *ACS Catal.* **8**, 2857-2863 (2018).
