# Supplementary material for: Electro-reduction of carbon dioxide at low over-potential at a metal–organic framework decorated cathode
Source: Nat Commun. 2020 Oct 29;11:5464. doi: 10.1038/s41467-020-19236-4 (PMC7596083; doi:10.1038/s41467-020-19236-4)
Supplement: Supplementary file 2 — Peer Review File [file 41467_2020_19236_MOESM2_ESM.pdf]

Reviewer #1 (Remarks to the Author):

Comments:
In this manuscript, the author fabricated a metal-organic framework decorated copper cathode for CO_2 electroreduction, namely the Cu2 (L)-e/Cu, which exhibited remarkable performance. The utilization of metal-organic frameworks as electrocatalyst is a less explored research area. However, the technical quality and discussion of mechanism need improvements. Therefore, I suggest major revision before publication in Nature Communications. The comments are as follows:
1. Cu catalysts are well known to produce unique C2+ products with low selectivity. So many researchers struggled to raise the FE of C2+ for Cu catalyst. The significance to produce formate with Cu catalysts should be rationalized and justified in the introduction part.
2. There are many literature reports on CO2RR to formate (for example, Sn-based and Bi-based catalysts). The authors are suggested to compare their catalyst with the state-of-the-art and justify their results. Besides, the description of the catalytic performance section compared with the state-of-the-art should be given as the more relevant data in charts or tables.
3. The article made some mistakes and errors. For example, the description of legend does not correspond to the figure. In figure 1, the scale bars of b, c and d are 300 um, 300 nm and 100 nm, respectively.
4. The catalyst was formed by electro-synthesis of the MOF on copper foam. However, the Cu foam itself already has been explored as electrocatalyst (ACS Catal. 2016, 6, 6, 3804.) and could produce a variety of products. In this case, the polycrystalline MOF coating could probably not cover the copper foam entirely. But why was formic acid the only carbon-containing product? This counterintuitive phenomenon and its mechanism should be throughout discussed, and the in-situ technology is probably needed to explore the “real” catalysts. The free Cu-foam electrode with FE_{HCOOH} of 20.5% should be discussed in the context of Cu2 (L)-e/Cu and Cu2 (L)-t/CP.
4. Furthermore, from the aspect of MOFs, Cu-carboxylate MOFs, such as HKUST-1 (J. Am. Chem. Soc. 2018, 140, 36, 11378) and 2-D Cu-MOF (Chem. Sci., 2019, 10, 2199) underwent the calcination or in-situ transformation to be the “catalyst” and enhance the stability in water or ionic liquid electrolytes. The real catalyst is not intact Cu-carboxylate MOF anymore in the previous two cases. In this paper, the active site was described as the coper center with uncoupled electron in Cu2(L), which was structurally unclear to the readers. Is it still MOF? Furthermore, why the decoration method could generate the highly active uncoupled electrons in MOFs? Please try to tune the number of uncoupled Cu (II) active sites in the Cu2(L)-e/Cu system and test the catalytic activity.
5 Is Cu2(L)-e/Cu still catalytically active in the water? Or other electrolyte systems.
6. The EPR measurement of the organic ligand, pure Cu foam and ionic liquid should be performed to check the background of EPR signal and give a fair comparison.
7. The quantities of uncoupled Cu (II) sites slightly decreased after a peak maximum (Figure 4a), which may be indicated the decomposition of catalysts. The long-term stability of the materials should be evaluated.
8. “The onset potential for reduction of CO2 to formic acid at 1.45 V vs Ag/Ag+, representing a record low value for electro-reduction of CO2 in an organic electrolyte.” should be revised. See Chem. Sci. 2019, 10, 2199.
9. Please provide the details about the simulation, especially the different structural models for Cu2(L)-e and Cu2(L)-t. The authors must consider the potential in-situ structural changes during electrocatalysis in ILs.

Reviewer #2 (Remarks to the Author):

General comments

The manuscript entitled "Electro-reduction of CO2 at low over-potential at a metal-organic framework decorated cathode" report the synthesis of a Cu-elcetrode. This decorated copper-based electrode has

a remarkable potential to start the reduction of CO2 to formic acid at -1.45 V compared to Ag/Ag+, which represents a record value for CO2 electro-reduction in an organic electrolyte compared to other studies in literature. These rather remarkable results in terms of conversion of CO2 to formic acid have been the subject of work in electrochemistry but also in EPR in order to understand the active sites that support the reactivity of the material. The authors thus showed unambiguously that this electroreductive activity is supported by monomeric copper defects while the binuclear sites are only slightly affected. These results are supported by a multi-frequency EPR approach (X-band and Q-band). I find it a pity that the extremely representative Figure S8 is not incorporated in the body of the paper. One of the very innovative aspects of this paper is to have combined a multi-technique experimental and theoretical approach leading to relevant and very convincing results.
From my point of view this paper is very innovative in the field of CO2 remediation by electrochemical techniques.

Détail comments

Page 3: The authors describe the binuclear system as strongly coupled by a strong antiferromagnetic exchange.
1- Did the authors measure this value experimentally by varying the temperature?
2- have the authors been able to observe the forbidden transition at half field as it is often the case for copper binuclear complexes?
3- In the simulation of the spectra, did the authors introduce this J value? If this is the case, the authors could add this parameter in the S2 tables.
Figure S19 :
1 -was this experiment carried out in operando where these measurements were made ex-situ. If they were carried out in opérando it would be interesting to mention it in the manuscript.
2- it is interesting to see that the EPR measurements are fully consistent with the progressive loss after 75 min of mononuclear Cu(II) sites and the XPS is interpreted as the formation of metallic copper. Did the authors try to detect the presence of this species by lowering the temperature for example, or by working at very low amplitude modulation and low microwave power.
Is it conceivable that the reduction of mononuclear sites is not done at 2 electrons but at 1 electron which would lead to the formation of Cu(I).
page7 : on the EPR analyses section, should provide the amplitude modulation used but also the code they have used for spectra simulation.

Conclusion

In conclusion, this work brings new results regarding CO2 electro-reduction and in particular the fact that only the mono nuclear species of copper are active. Moreover, these electro-synthetized materials have a remarkably low potential compared to what is described in the current literature. For all these reasons I think that this article has its place to be published in Nature Communications which will allow a wide dissemination of these results and should stimulate research on CO2 electro-reduction based on such electrode materials.

Reviewer #3 (Remarks to the Author):

The authors report a presumably successful synthesis for creating a defect-rich MOF catalyst for CO2 reduction to formic acid with low overpotential. The authors argue the high activity is driven by coordinatively unsaturated copper centers in the defect-rich catalyst. However, a convincing causal link between higher concentration of unsaturated Cu sites and activity is lacking; the obvious induction period of the catalyst and the co-evolution of Cu(0) along with uncoupled Cu(II) points to at least several alternative hypotheses for the active site besides the uncoupled Cu(II). Furthermore, the DFT calculations are incomplete and fail to support the argument of CO2 reduction on the uncoupled Cu(II)

sites. In light of these concerns regarding the conclusions of the paper I would not recommend publishing in Nature Communications.

Some specific comments below:

1. Figures showing the DFT geometries of the intermediates from CO2 (adsorbed) to formic acid should be supplied in the SI.

2. The hydrogen binding free energies must be explicitly calculated on the same site to verify the the similar suppression of HER experimentally.

3. Generally the path to produce formate is via a HCOO intermediate, not a COOH intermediate, as shown by many theoretical studies on copper (COOH leads to the formation of CO instead). At the very least, both HCOO and COOH pathways should be calculated on the pristine and defect sites. In addition the path to form CO should also be considered as a comparison. There are many references regarding this, to name a few: ACS Catal. 2017, 7, 7, 4822–4827; J. Phys. Chem. Lett. 2015, 6, 20, 4073–4082

4. The binding free energy of COOH is hard to rationalize in terms of the experimental performance. The binding energy appears to be very positive at 2-2.5 eV. This seems unreasonably high (and would point to a very high overpotential) and suggests these sites are not actually active for CO2 reduction. A comparison with the pure Cu surface should also be provided using the same computational method (e.g. in a recent DFT work here: J. Phys. Chem. C 2018, 122, 21, 11392–11398. the values are much lower in energy on metallic copper.)

5. Solvation effects are completely omitted in either the calculation or discussion.

We thank the reviewers for their constructive comments and our responses are given below in bold italics. We attach files for the revised manuscript and SI with all changes highlighted in yellow for clarity and have also submitted clean versions of both.

Reviewer #1 (Remarks to the Author):
Comments:
In this manuscript, the author fabricated a metal-organic framework decorated copper cathode for CO_2 electroreduction, namely the Cu_2(L)-e/Cu, which exhibited remarkable performance. The utilization of metal-organic frameworks as electrocatalyst is a less explored research area. However, the technical quality and discussion of mechanism need improvements. Therefore, I suggest major revision before publication in Nature Communications. The comments are as follows:

1. Cu catalysts are well known to produce unique C2+ products with low selectivity. So many researchers struggled to raise the FE of C2+ for Cu catalyst. The significance to produce formate with Cu catalysts should be rationalized and justified in the introduction part.
Revised. Additional discussions have been added to the introduction on page 1.

2. There are many literature reports on CO_2RR to formate (for example, Sn-based and Bi-based catalysts). The authors are suggested to compare their catalyst with the state-of-the-art and justify their results. Besides, the description of the catalytic performance section compared with the state-of-the-art should be given as the more relevant data in charts or tables.
Revised. A comprehensive comparison with literature examples is now given in Supplementary Table 3.

3. The article made some mistakes and errors. For example, the description of legend does not correspond to the figure. In figure 1, the scale bars of b, c and d are 300 um, 300 nm and 100 nm, respectively.
Corrected. We apologise for this typo.

4. The catalyst was formed by electro-synthesis of the MOF on copper foam. However, the Cu foam itself already has been explored as electrocatalyst (ACS Catal. 2016, 6, 6, 3804.) and could produce a variety of products. In this case, the polycrystalline MOF coating could probably not cover the copper foam entirely. But why was formic acid the only carbon-containing product? This counterintuitive phenomenon and its mechanism should be throughout discussed, and the in-situ technology is probably needed to explore the "real" catalysts. The free Cu-foam electrode with FE_{HCOOH} of 20.5% should be discussed in the context of Cu_2(L)-e/Cu and Cu_2(L)-t/CP.
This is an excellent point. The Cu-foam reported in ACS Catal. was prepared by a template-assisted electrodeposition methods, and shows a nanostructured surface, which plays a key role in the production of various C-containing products. The Cu-foam used in this work is not decorated with any nanostructure and has a smooth surface as shown in Fig. 1. We have added this reference and revised the manuscript accordingly to clarify this point on page 5.

4. Furthermore, from the aspect of MOFs, Cu-carboxylate MOFs, such as HKUST-1 (J. Am. Chem. Soc. 2018, 140, 36, 11378) and 2-D Cu-MOF (Chem. Sci., 2019, 10, 2199) underwent the calcination or in-situ transformation to be the "catalyst" and enhance the stability in water or ionic liquid electrolytes. The real catalyst is not intact Cu-carboxylate MOF anymore in the previous two cases. In this paper, the active site was described as the copper center with uncoupled electron in Cu_2(L), which was structurally unclear to the

readers. Is it still MOF? Furthermore, why the decoration method could generate the highly active uncoupled electrons in MOFs? Please try to tune the number of uncoupled Cu (II) active sites in the Cu_2(L)-e/Cu system and test the catalytic activity.

The above references have been added as refs 18 and 17, respectively. In our work, Cu-MOFs were prepared by solvothermal reactions or by electro-synthesis without post-synthetic treatment other than what may happen on electro-reduction of CO_2. The Cu-foam anode was covered rapidly by particles of Cu_2(L)-e during the electro-synthesis over a 10 minute period, and the Cu(II) defect sites were produced by the rapid MOF synthesis on the Cu(II)-rich surface. It is difficult to control precisely the number of uncoupled Cu(II) sites because the electro-synthesis is completed as the surface of the Cu-foam becomes fully covered with MOF particles and as the supply of Cu^{2+} ions from the anode for further MOF growth is cut off. Nonetheless, we have determined by EPR spectroscopy that the Cu_2(L)-t and Cu_2(L)-e electrodes contain 1.5% and 15.3% of uncoupled Cu(II) sites, respectively (page 4). This observation is consistent with the enhanced catalytic activity of the latter. In addition, EPR spectra of Cu_2(L)-t and Cu_2(L)-e (Supplementary Figs. 7, 8 and Supplementary Table 2) display spectral features that are consistent with those of copper acetate (Ref. 36) and other $[Cu_2(OOCR)_4]$ paddlewheel complexes (Ref. 50), confirming that the structure of the MOF node is preserved. PXRD confirms that the structure of the MOF remain intact. The uncoupled Cu(II) component also gives an axial EPR spectrum ($g_{zz} >> g_{xx}=g_{yy}$; $A_{zz}>>A_{xx}=A_{yy}$), and we therefore been able to ascertain the % levels of coupled and uncoupled Cu(II) centres in these materials. Moreover, new DFT calculations have been conducted to gain insights into the mechanism of electrocatalysis. Additional results and analysis have been added to the revised manuscript notably on page 6 and 7, Figure 2 of the manuscript and pages S21-S52, new Supplementary Tables 4-12 and new Supplementary Figs 5, 25-28 in SI.

We could certainly seek to alter and tune the % of uncoupled Cu(II) sites but this would require further experiments and the development and modification of the current synthetic method, which is consistent and reliable.

As an aside, turning a strongly antiferromagnetic system with an exchange coupling ~ 300 cm^{-1} that contains four carboxylate bridging ligands into a ferromagnetic species (thus mimicking an uncoupled Cu(II) site) that would have the exact spectral parameters of the excited S = 1 spin state of the $[Cu_2(OOCR)_4]$ systems is highly unlikely.

5. Is Cu_2(L)-e/Cu still catalytically active in the water? Or other electrolyte systems.

Unfortunately, Cu_2(L)-e/Cu is not stable in water. This is consistent with reported results (ref. 15) that Cu-MOFs generally have poor stability in aqueous electrolyte for CO_2 reduction and decompose to copper oxides rapidly. This has been added to page 5.

6. The EPR measurement of the organic ligand, pure Cu foam and ionic liquid should be performed to check the background of EPR signal and give a fair comparison.

Revised. These spectra have been added to the revised manuscript (Supplementary Figs. 5, 6, 19).

7. The quantities of uncoupled Cu (II) sites slightly decreased after a peak maximum (Figure 4a), which may be indicated the decomposition of catalysts. The long-term stability of the materials should be evaluated.

Revised. The reviewer is correct. There is some loss of uncoupled Cu(II) after ca 1.5h for Cu_2(L)-e/Cu but this is far less than for Cu_2(L)-t/CP or HKUST-1-e/Cu. However, Cu_2(L)-e/Cu exhibits long-term

electrochemical stability and the catalysis is stable as shown by Supplementary Figs. 21, 22 and 23.

8. "The onset potential for reduction of CO_2 to formic acid at −1.45 V vs Ag/Ag+, representing a record low value for electro-reduction of CO_2 in an organic electrolyte." should be revised. See Chem. Sci. 2019, 10, 2199.
Revised. This reference has been cited as ref 17 and text modified on page 5.

9. Please provide the details about the simulation, especially the different structural models for Cu_2(L)-e and Cu_2(L)-t. The authors must consider the potential in-situ structural changes during electrocatalysis in ILs.
Revised. The structural model and atomic coordinates for all intermediates used in the DFT calculations have been added to the SI (page 21 – 52). We agree entirely with the reviewer that there may well be potential in situ structural changes during electrocatalysis. However, the exact nature of this can hardly be predicted or modelled accurately by DFT. The potential impact of structural change of electrodes has been added in the revised manuscript on pages 6 and 7, and the new ref 42 highlights reduction of Cu(II) to Cu(I) in a paddlewheel MOF with concomitant distortion of the coordination sphere.

Reviewer #2 (Remarks to the Author):

The manuscript entitled "Electro-reduction of CO_2 at low over-potential at a metal-organic framework decorated cathode" report the synthesis of a Cu-elcetrode. This decorated copper-based electrode has a remarkable potential to start the reduction of CO_2 to formic acid at -1.45 V compared to Ag/Ag+, which represents a record value for CO_2 electro-reduction in an organic electrolyte compared to other studies in literature. These rather remarkable results in terms of conversion of CO_2 to formic acid have been the subject of work in electrochemistry but also in EPR in order to understand the active sites that support the reactivity of the material. The authors thus showed unambiguously that this electroreductive activity is supported by monomeric copper defects while the binuclear sites are only slightly affected. These results are supported by a multi-frequency EPR approach (X-band and Q-band). I find it a pity that the extremely representative Figure S8 is not incorporated in the body of the paper. One of the very innovative aspects of this paper is to have combined a multi-technique experimental and theoretical approach leading to relevant and very convincing results. From my point of view this paper is very innovative in the field of CO_2 remediation by electrochemical techniques.
Supplementary Fig. 8 has been moved to the main text (Figs. 2h, 2i). This makes Figure 2 rather large and complex but given the on line nature of the journal this is acceptable in our view.

Page 3: The authors describe the binuclear system as strongly coupled by a strong antiferromagnetic exchange.

1- Did the authors measure this value experimentally by varying the temperature?

The magnetic behavior of copper(II) acetate and related Cu(II) paddlewheel systems is well known, and is marked by strong antiferromagnetic coupling between Cu(II) ions leading to a well isolated singlet (S = 0) ground state. The singlet-triplet energy gap has been measured by INS (298 cm^{-1}; H Güdel, Inorg. Chem. 1979, 18, 1021), and magnetic susceptibility studies (315 cm^{-1}; ref. 34). The observed EPR spectra at room temperature are due to the excited triplet (S = 1) spin state, and have very specific g, D, E characteristics: $g_z = 2.35 \pm 0.03$, $g_{x,y} = 2.07 \pm 0.03$, $D = 0.34 \pm 0.03\ cm^{-1}$, and E~0, and display a 'half-field' forbidden transition (ms =± 2). Our recorded spectra are typical for such a Cu(II) paddlewheel structure (ref. 50). In

this work, we conducted electro-reduction of CO_2 at room temperature, and conducted EPR studies at the same temperature aiming to monitor changes in MOF structure during the electrolysis.

2- have the authors been able to observe the forbidden transition at half field as it is often the case for copper binuclear complexes?

We observed the forbidden transition at ~5300 G in Fig. 2g and Supplementary Figs. 8a, 8b, confirming the S = 1 spin state of a binuclear Cu_2 entity. We have added this to the text on page 3.

3- In the simulation of the spectra, did the authors introduce this J value? If this is the case, the authors could add this parameter in the S2 tables.

Revised. Simulation of the EPR spectra of S = 1 species involved a spin Hamiltonian that included the axial and rhombic zero-field splitting terms (D and E) in order to compare the results to known paddlewheel systems. We have added the equation of the Spin Hamiltonian in the main text and provided additional information on the modelling of EPR data on page 8 of the manuscript.

Figure S19 :

1 -was this experiment carried out in operando where these measurements were made ex-situ. If they were carried out in operando it would be interesting to mention it in the manuscript.

The experiment is not in operando. The electrodes were taken out of the electrochemical cell and EPR spectra of the electrodes were collected every 15 minutes during electrolysis at −1.8 V vs. Ag/Ag^+, taking care to maintain the experimental conditions unchanged. This has been added to the legend for Supplementary Fig. 19

2- it is interesting to see that the EPR measurements are fully consistent with the progressive loss after 75 min of mononuclear Cu(II) sites and the XPS is interpreted as the formation of metallic copper. Did the authors try to detect the presence of this species by lowering the temperature for example, or by working at very low amplitude modulation and low microwave power.

Is it conceivable that the reduction of mononuclear sites is not done at 2 electrons but at 1 electron which would lead to the formation of Cu(I).

page7 : on the EPR analyses section, should provide the amplitude modulation used but also the code they have used for spectra simulation.

Revised. We have concluded that formation of diamagnetic Cu(I) ($3d^{10}$) is more plausible, and have revised the manuscript accordingly. This assignment is supported by Cu2p XPS data and Auger spectra (new Supplementary Fig. 20), with additional ref 42 supporting Cu(I) formation. Amplitude modulation and code used for EPR simulation have been added to the revised manuscript on page 8 of the manuscript.

In conclusion, this work brings new results regarding CO_2 electro-reduction and in particular the fact that only the mono nuclear species of copper are active. Moreover, these electro-synthetized materials have a remarkably low potential compared to what is described in the current literature. For all these reasons I think that this article has its place to be published in Nature Communications which will allow a wide dissemination of these results and should stimulate research on CO_2 electro-reduction based on such electrode materials.

Reviewer #3 (Remarks to the Author):

The authors report a presumably successful synthesis for creating a defect-rich MOF catalyst for CO_2 reduction to formic acid with low overpotential. The authors argue the high activity is driven by coordinatively unsaturated copper centers in the defect-rich catalyst. However, a convincing causal link between higher concentration of unsaturated Cu sites and activity is lacking; the obvious induction period of the catalyst and the co-evolution of Cu(0) along with uncoupled Cu(II) points to at least several alternative hypotheses for the active site besides the uncoupled Cu(II). Furthermore, the DFT calculations are incomplete and fail to support the argument of CO_2 reduction on the uncoupled Cu(II) sites. In light of these concerns regarding the conclusions of the paper I would not recommend publishing in Nature Communications.
Additional EPR experiments and DFT calculations have been conducted to strengthen the link between active Cu sites and catalytic activity. Please see below.

Some specific comments below:
1. Figures showing the DFT geometries of the intermediates from CO_2 (adsorbed) to formic acid should be supplied in the SI.
Revised. These have been added in SI.

2. The hydrogen binding free energies must be explicitly calculated on the same site to verify the the similar suppression of HER experimentally.
Revised. This has been added to the revised manuscript on page 5 and Supplementary Fig. 28.

3. Generally the path to produce formate is via a HCOO intermediate, not a COOH intermediate, as shown by many theoretical studies on copper (COOH leads to the formation of CO instead). At the very least, both HCOO and COOH pathways should be calculated on the pristine and defect sites. In addition the path to form CO should also be considered as a comparison. There are many references regarding this, to name a few: ACS Catal. 2017, 7, 7, 4822–4827; J. Phys. Chem. Lett. 2015, 6, 20, 4073–4082
Revised. Both O-bound HCOO and C-bound COOH are plausible intermediates in the generation of formic acid (refs. 20, 21). We agree with the reviewer that an O-bound HCOO intermediate would lead to the formation of formic acid only. As suggested by the referee, DFT calculations via both O-bound HCOO and C-bound COOH pathways have been conducted (Supplementary Fig. 25-27). The formation of O-bound HCOO is more facile over both pristine and defect $Cu_2(L)$ than formation of C-bound COOH. Particularly, defect $Cu_2(L)$ has a much lower energy barrier for the formation of O-bound HCOO, resulting in the production of formic acid. This also excludes the possibility of CO or other C-containing products being formed due to the pathway that requires C-bound COOH being less favoured (Supplementary Fig. 25). This discussion has been added to the revised manuscript (pages 6-7) and the two references cited above have been added as refs 46 and 47.

4. The binding free energy of COOH is hard to rationalize in terms of the experimental performance. The binding energy appears to be very positive at 2-2.5 eV. This seems unreasonably high (and would point to a very high overpotential) and suggests these sites are not actually active for CO_2 reduction. A comparison with the pure Cu surface should also be provided using the same computational method (e.g. in a recent DFT work here: J. Phys. Chem. C 2018, 122, 21, 11392–11398. the values are much lower in energy on metallic copper.)
Both O-bound HCOO and C-bound COOH are plausible intermediates. We have calculated these two

pathways over pristine and defect $Cu_2(L)$ electrodes. Pristine $Cu_2(L)$ shows higher binding free energy over these two pathways, which suggests indeed a low activity for CO_2 reduction. Defect $Cu_2(L)$ has an evidently lower free energy toward the O-bound HCOO pathway, which is comparable with other efficient electrodes. Pure copper has a low binding energy and is considered as an effective electrode for CO_2 reduction. The DFT analysis reported in the above reference (ref. 48 in the revised manuscript) has confirmed that pure copper is prone to generate both C-bound COOH and O-bound HCOO intermediates, yielding various C-containing products. In this work, formic acid was the only product from CO_2, and therefore we believe Cu^{2+} is the active sites in $Cu_2(L)$. Additional DFT analysis and discussions have been added to the revised manuscript.

5. Solvation effects are completely omitted in either the calculation or discussion.
This is an excellent point. Solvation effects have been considered for both CO_2 reduction and the hydrogen evolution reaction. Lower Gibbs free energies are observed after considering solvation effects for both pristine and defect $Cu_2(L)$ via different pathways for CO_2 reduction (new Supplementary Fig. 27). For hydrogen evolution solvation effects are less dramatic (new Supplementary Fig. 28). This has been added to the revised manuscript.

Reviewer #1 (Remarks to the Author):

The authors have addressed all my concerns. This manuscript is ready for acceptance.

Reviewer #2 (Remarks to the Author):

In this revised version of the manuscript the authors have provided explanations and clarified the EPR experiences with modifications and clarifications at the manuscript level. Even if this system is well described in the literature, the fact of having made the measurements at two frequencies brings a plus at the level of redox characterization and structural changes.
The authors have taken my remarks into account and have modified the manuscript and the additional material part accordingly.
With regard to the other remarks made by the two other reviewers, they have also provided me with the elements of response in the cover letter and also modified the text accordingly.
In conclusion, the results obtained with this material for CO_2 electroreduction are very convincing with a remarkable performance. I believe that this work is well worth publishing in the journal Nature Communications.

Reviewer #3 (Remarks to the Author):

The authors have made some efforts to improve the theoretical portion of the work including the calculation of additional intermediates and inclusion of solvation. This does seem to be a step in the right direction and the energies for formic acid formation via HCOO appear to be more reasonable than the previously presented path. It also does appear the defect MOF (model) is slightly more selective to formic acid over CO. However compared to other systems the energetics for formic acid formation are not remarkably favorable either. There are still reservations on the connection to the experimental system, and the validity of the model being studied. At best, the results are qualitatively consistent with the overall conclusion that defects in these MOF catalysts can improve performance for CO2RR.

We thank the reviewers for their constructive comments and our responses are given below in bold italics. We attach files for the revised manuscript and SI with all changes highlighted in yellow for clarity and have also submitted clean versions of both.

Reviewer #1 (Remarks to the Author):
The authors have addressed all my concerns. This manuscript is ready for acceptance.
We thank the reviewer.

Reviewer #2 (Remarks to the Author):
In this revised version of the manuscript the authors have provided explanations and clarified the EPR experiences with modifications and clarifications at the manuscript level. Even if this system is well described in the literature, the fact of having made the measurements at two frequencies brings a plus at the level of redox characterization and structural changes.
The authors have taken my remarks into account and have modified the manuscript and the additional material part accordingly.
With regard to the other remarks made by the two other reviewers, they have also provided me with the elements of response in the cover letter and also modified the text accordingly.
In conclusion, the results obtained with this material for CO2 electroreduction are very convincing with a remarkable performance. I believe that this work is well worth publishing in the journal Nature Communications.
We thank the reviewer.

Reviewer #3 (Remarks to the Author):
The authors have made some efforts to improve the theoretical portion of the work including the calculation of additional intermediates and inclusion of solvation. This does seem to be a step in the right direction and the energies for formic acid formation via HCOO appear to be more reasonable than the previously presented path. It also does appear the defect MOF (model) is slightly more selective to formic acid over CO. However compared to other systems the energetics for formic acid formation are not remarkably favorable either. There are still reservations on the connection to the experimental system, and the validity of the model being studied. At best, the results are qualitatively consistent with the overall conclusion that defects in these MOF catalysts can improve performance for CO2RR.
Revised. We agree with the reviewer that the energetics for formic acid formation are not remarkably favorable compared with other systems. From the DFT calculations, Cu_2(L)-e/Cu shows a lower Gibbs free energy due to its defect structure with a high concentration of uncoupled Cu(II) centres (Fig. 4f). This indicates that the more defects, the lower the Gibbs free energy. Before electrolysis, Cu_2(L)-t and Cu_2(L)-e show 1.5% and 15.3% uncoupled Cu(II) sites, respectively, and in the DFT calculations pristine and defect Cu_2(L) models represent the original Cu_2(L)-t and Cu_2(L)-e, respectively. However, both pristine and defect electrodes show an increase in uncoupled Cu(II) content during electrolysis (Fig. 4a) indicating that more active sites are generated, consistent with the increasing current density for CO_2 reduction observed over the first hour of electrolysis. Text on this has been added to the revised manuscript.
